# Molecular analyses of circadian gene variants reveal sex-dependent links between depression and clocks

**DOI:** 10.1038/tp.2016.9

**Published:** 2016-03-01

**Authors:** S-q Shi, M J White, H M Borsetti, J S Pendergast, A Hida, C M Ciarleglio, P A de Verteuil, A G Cadar, C Cala, D G McMahon, R C Shelton, S M Williams, C H Johnson

**Affiliations:** 1 Department of Biological Sciences, Vanderbilt University, Nashville, TN, USA; 2 Department of Medicine, University of California San Francisco, San Francisco, CA, USA; 3 Instituto de Estudios Celulares, Genéticos y Moleculares, Universidad Nacional de Jujuy, Jujuy, Argentina; 4 Department of Biology, University of Kentucky, Lexington, KY, USA; 5 Department of Psychophysiology, National Institute of Mental Health, Tokyo, Japan; 6 Department of Neuroscience, Brown University, Providence, RI, USA; 7 Department of Molecular Physiology and Biophysics, Vanderbilt University School of Medicine, Nashville, TN, USA; 8 Department of Psychiatry, University of Alabama School of Medicine, Birmingham, AL, USA; 9 Department of Epidemiology and Biostatistics, Case Western Reserve University, Cleveland, OH, USA

## Abstract

An extensive literature links circadian irregularities and/or sleep abnormalities to mood disorders. Despite the strong genetic component underlying many mood disorders, however, previous genetic associations between circadian clock gene variants and major depressive disorder (MDD) have been weak. We applied a combined molecular/functional and genetic association approach to circadian gene polymorphisms in sex-stratified populations of control subjects and case subjects suffering from MDD. This approach identified significant sex-dependent associations of common variants of the circadian clock genes *hClock*, *hPer3* and *hNpas2* with major depression and demonstrated functional effects of these polymorphisms on the expression or activity of the hCLOCK and hPER3 proteins, respectively. In addition, hCLOCK expression is affected by glucocorticoids, consistent with the sex-dependency of the genetic associations and the modulation of glucocorticoid-mediated stress response, providing a mechanism by which the circadian clock controls outputs that may affect psychiatric disorders. We conclude that genetic polymorphisms in circadian genes (especially hClock and hPer3, where functional assays could be tested) influence risk of developing depression in a sex- and stress-dependent manner. These studies support a genetic connection between circadian disruption and mood disorders, and confirm a key connection between circadian gene variation and major depression.

## Introduction

Circadian rhythms pervasively control the behavior, physiology and biochemistry of humans, including the timing of sleep. Circadian irregularities and/or sleep abnormalities have been linked to mood disorders such as major depressive disorder (MDD), bipolar disorder and seasonal affective disorder by studies of jet lag, sleep deprivation, chronotype, stress and comorbidity of mood disorders with advanced or delayed sleep phase syndrome.^1, 2, 3, 4, 5, 6^ Indeed, altered sleep/wake cycles are a critical feature for diagnosis of many mood disorders in the Diagnostic and Statistical Manual of Mental Disorders, Fifth Edition. Several effective pharmacological and non-pharmacological therapies for mood disorders, including bright light, sleep deprivation and/or phase-resetting paradigms, appear to function by modulating circadian parameters.^3, 4, 6, 7^ Moreover, a recent study of postmortem human brains reported that daily rhythmic patterns of gene expression in the brain are seriously disrupted and/or desynchronized in MDD subjects,^8^ further emphasizing the correlation between abnormal circadian rhythms and the risk and severity of mood disorders.

There is also a strong genetic component underlying many mood disorders,^9, 10^ and there have been a few tantalizing suggestions of links between clock gene polymorphisms and bipolar disorder.^11, 12, 13^ Nevertheless, most association studies to date have concluded that genetic associations between circadian clock gene variants and MDD are weak at best.^4, 11, 12, 13, 14, 15^ However, in psychiatric disorders such as MDD, bipolar disorder, schizophrenia and so on, phenotyping the overt manifestations of the disorder may lead to misleading interpretations of etiology. In other words, overlapping and/or common symptoms for a psychiatric disorder may group syndromes that do not have a common mechanistic cause. Therefore, interpreting diagnoses/phenotypes can be problematic for genetic studies of psychiatric disorders, especially in meta-analyses where the standard of phenotyping may be very different among studies. Moreover, the previous genome-wide association studies (GWAS) might have missed significant associations because GWAS studies often include so many subjects and phenotypes that significant associations and biology can be lost in multiple testing corrections. In addition, several disorders associated with disruptions in circadian systems, including MDD, display significant disparities in prevalence and/or severity between the sexes.^16^ This could be, in part, due to sex-specific genetic effects that have not been generally explored in GWAS studies.

The process of re-examining the molecular impact of a polymorphism in the three-prime untranslated region (3′-UTR) of the human CLOCK protein led us to a re-evaluation of the published GWAS studies for clock gene polymorphisms in association with MDD. We found significant sex-dependent associations of MDD with the circadian genes *hClock*, *hPer3* and *hNpas2*. In the currently accepted molecular model of the circadian clock,^17^ positive and negative core clock proteins form an ~24-h feedback loop. Two bHLH-PAS transcription factors, CLOCK and ARNTL1/2 (aka mBMAL1/2 in mouse), heterodimerize and act as positive elements to initiate transcription from genes containing E-box *cis*-regulatory elements, including the Per-1/2/3 and Cry-1/2 genes.^18, 19, 20^ PER and CRY proteins heterodimerize and subsequently repress transcription of their own (and other) genes by directly inhibiting CLOCK/ARNTL.^18, 19, 20^ Therefore, PER-1/2/3 and CRY-1/2 function as negative components in the transcription/translation feedback loop. In some parts of the brain (especially forebrain), neuronal PAS domain protein 2 (NPAS2) appears to be an alternative key partner with ARNTL.^21, 22^ The CLOCK(NPAS2)/ARNTL complex regulates rhythmic transcription of clock-controlled genes in tissues throughout the body, and this includes at least 15% of all mammalian transcripts;^23, 24^ many of these gene expression patterns are disrupted in humans suffering from MDD.^8^ Our data indicate that polymorphisms in *hClock* and *hPer3* genes impact the functional activity of these proteins and, together with a polymorphism in *hNpas2*, exhibit sex- and stress-dependent association with MDD in humans.

## Materials and methods

### Human subjects

In an effort to aid in the advancement of genetic research directed at severe neuropsychiatric disorders, the National Institute of Mental Health (NIMH) funded the Human Genetics Initiative (see full acknowledgments in the Supplementary Information).^25^ The Human Genetics Initiative compiled a database of clinically diagnosed MDD pedigrees, including MDD cases and affected relatives (Depression 2.0, NIMH Major Depression Genetics Initiative). All the participants were administered the Diagnostic Interview for Genetic Studies (DIGS v2.0) by trained interviewers. Probands who self-reported as Caucasian and carried a clinical diagnosis of subtype 1 MDD, also termed melancholia, were selected from this data set for our study. The Human Genetics Initiative also collected a broad-based control sample to aid in case–control association studies focused on neuropsychiatric disorders. These controls were phenotyped using an online lifetime version of the Composite Instrument for Diagnostic Interviewing Short Form; an online clinical self-report assessment of MDD and several other psychiatric conditions that have been previously described.^26^ Informed consent was obtained for all Human Genetics Initiative data set and NIMH Major Depression Genetics Initiative participants before DNA collection. Further information is provided in the Supplementary Materials and Methods.

A diagnosis of subtype 1 MDD (melancholia) was used to define MDD case status; participants with no evidence of any form of MDD, either by clinical diagnosis or via targeted survey questions, were labeled study controls. After quality control procedures, 592 MDD cases and 776 MDD controls remained for analyses (further described in Supplementary Materials and Methods and in demographic information of Supplementary Tables S1A and S1B).

### Genotyping and association analyses

Samples were genotyped for a total of 32 genetic variants from eight genes. These markers were chosen based on their common frequency in the Caucasian population and/or their potential functional significance based on well-characterized roles in regulating circadian rhythms and/or sleep disorders.^27, 28^ After genotyping, the samples were assessed for genotyping efficiency (single-nucleotide polymorphism (SNP) and sample level) and deviation from Hardy–Weinberg equilibrium as measures of quality control. Hardy–Weinberg equilibrium was assessed in case and control samples separately. The following quality control exclusion criteria were applied to the data set: (1) SNP genotyping efficiency <95% and (2) sample genotyping efficiency <95%, Hardy–Weinberg equilibrium test *P*-value <0.001. All quality control testing was performed using PLINK.^29^ After quality control, 18 genetic variants remained for analysis in our data set. Before beginning analysis, we performed power calculations using PS Power^30^ to determine whether our study was adequately powered to detect common variants with modest effects on MDD. *A priori* power calculations indicated that our study was well powered (⩾80%) to detect common variants (minor allele frequency ⩾0.2) with modest effect sizes, and *α*=0.5 (Supplementary Figure S1).

To assess statistical association between the analyzed SNPs and MDD, single locus analyses were performed in the combined data set (*n*=592 cases, 776 controls). Allelic and genotypic chi-square analyses were performed, using PLINK.^29^ Where appropriate, Fisher's exact test of association was performed. To investigate the possibility of potentially differing effects of the analyzed SNPs on MDD between the genders, sex-separated analyses were also performed (males, *n*=94 cases, 253 controls; females, *n*=498 cases, 523 controls) for all markers that showed a *P*-value <0.2 in chi-square genotypic or allelic tests in the full data set. Correction for multiple testing of chi-square *P*-values was performed using false discovery rate (*q*=0.1).^31^ An alternative single locus analysis, prevalence-based association testing, based on the principles of Hardy–Weinberg equilibrium has been shown in simulation studies to be more powerful than chi-square testing under some genetic models and was also performed to provide independent evidence for association.^32^ Association analysis methods are described fully in the Supplementary Materials and Methods.

### Functional analyses

The P_SV40_::FLuc::3′-UTR firefly luciferase reporter for the 3′-UTR of *hClock* (containing the major allele T) was provided by Dr Malcolm von Schantz.^33^ To create a minor allele reporter, a T to C mutation at position 3111 (rs1801260) was introduced by site-directed mutagenesis (Stratagene, San Diego, CA, USA) and verified by sequencing. To create P_Clock_::FLuc plasmids so that the luciferase expression is under the control of the endogenous *hClock* promoter, a fragment extending from the *hClock* upstream region (−1908 from the transcription start site) to the first intron (+101 from the transcription start site) was amplified by PCR from human cell genomic DNA and was used to replace the SV40 promoter of the P_SV40_::FLuc-T/C plasmids. The *hPer3* expression plasmid under the control of the CMV promoter (P_CMV_::hPer3) was provided by Dr Joon-Kyu Lee. A G to C mutation (rs228697) in the coding region of *hPer3* was introduced to change the proline at position 856 to an alanine residue by site-directed mutagenesis (Stratagene) that was verified by sequencing. Cell culture, immunoprecipitation and functional assay methods are fully described in the Supplementary Materials and Methods.

### Statistical analyses for functional assays

The statistical analyses for functional assays were performed by two-tail unpaired *t*-test, by one-way analysis of variance with Tukey *post hoc* or by two-way analysis of variance with Bonferroni *post hoc* test where appropriate. Welch's correction for unequal variances was applied to *t*-tests. Details of statistical analyses used for particular experiments are described in the corresponding figure legends. For more information, see Supplementary Materials and Methods.

## Results

### Molecular analyses of *hClock* variant shows enhanced transcriptional activity

A common polymorphism in *hClock* located in the 3′-UTR of the gene (rs1801260) has been associated with chronotype.^34^ The major allele at this site (position 3111 of *hClock*) is a T nucleotide and the minor allele is a C. A sequence analysis predicts that this variant will have significant effects on the secondary structure of the *hClock* transcript (analysis not shown). However, in a previous study of potential functional impact of this polymorphism, a reporter composed of firefly luciferase (FLuc) driven by the SV40 promoter and linked to the 3′-UTR of *hClock* with and without the rs1801260 SNP was tested in Cos-1 cells and the polymorphism was found to have no significant effect upon expression/turnover of the FLuc::3′-UTR fusion construct.^33^ We reasoned that this result might have been due to the cell type used for that test, namely Cos-1 (monkey kidney) cells. Because the expression of the proteome can vary significantly among mammalian cell lines, tissues and species, and the expression/turnover of molecular circadian clock components could be strongly influenced by the expression of other proteins, reporter constructs with human variants should be tested in a variety of cell lines with special focus upon cell lines derived from humans.

**Figure 1:**
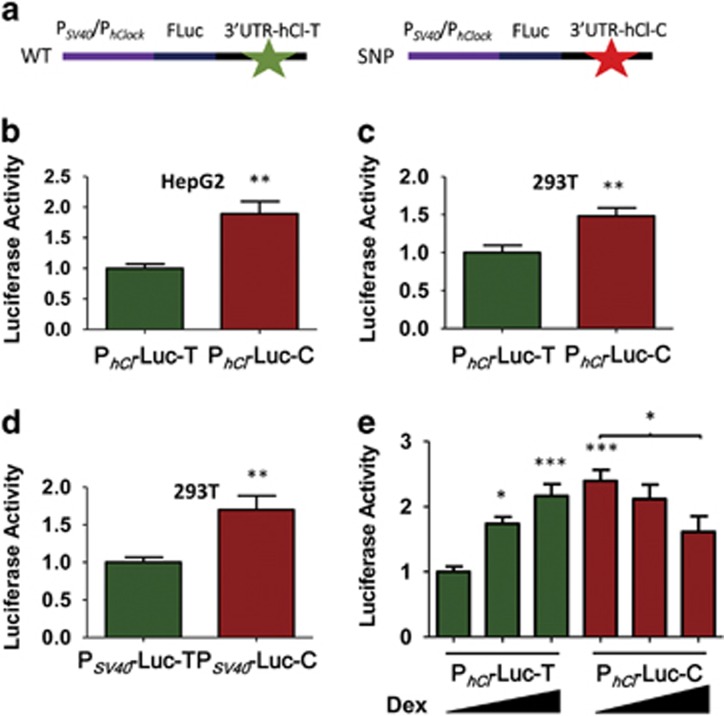
Functional analysis of *hClock* SNP in cell cultures. (**a**) Schematic shows the plasmids containing firefly luciferase (FLuc) gene and 3′-UTR of Clock with or without the rs1801260 SNP (SNP is ‘C' at position 3111 whereas the common allele is ‘T'). Two promoters were used to drive FLuc::3′-UTR expression: P_hClock_ includes 102 bp of *hClock*'s 5′-UTR that is downstream of the transcriptional start site, whereas P_SV40_ does not include any 5′-UTR. (**b**) HepG2 (human liver) cells transfected with P_hClock_::FLuc::3′-UTR. (**c**) 293 T (human kidney) cells transfected with P_hClock_::FLuc::3′-UTR. (**d**) 293 T (human kidney) cells were transfected with P_SV40_::FLuc::3′-UTR. For **b**–**d**, levels of FLuc activity were normalized to efficiency of transfection with co-transfected *Renilla* luciferase reporter (P_CMV_::RLuc). Activity of samples transfected with the reporter constructs containing the common 3′-UTR allele (Pclock-Luc-T or Psv40-luc-T) was set as 1.0. Results are shown as mean±s.e.m. of two (left panel, total *n*=10) or four (middle and right panels, total *n*=20) independent experiments with multiple repeats. **P<0.01 by two-tailed unpaired *t*-test. (**e**) Expression of *hClock* is modulated by the glucocorticoid dexamethasone (Dex) and the rs1801260 SNP. HepG2 (human liver) cells were transfected with P_hClock_::FLuc::3′-UTR with or without the rs1801260 SNP (*n*=12 from three independent experiments) and exposed to Dex at concentrations of 0, 50 and 500 nm for 2 h, starting at 22 h after transfection. *P<0.05, **P<0.01 and ***P<0.001 by one-way analysis of variance with Tukey *post hoc*. SNP, single-nucleotide polymorphism; UTR, untranslated region.

We therefore re-tested the potential effect of the 3111C SNP with a FLuc reporter (1) in a variety of cell lines derived from humans and (2) where the 3′-UTR was coupled with the promoter region of *hClock*, including 102 bp of its 5′-UTR (as compared with the originally tested P_SV40_ construct^33^ (Figure 1a). This re-examination revealed that the P_hClock_::FLuc::3′-UTR reporter exhibited significantly higher expression of FLuc protein when it is coupled to the 3′-UTR bearing the minor allele. Figures 1b and c depict expression results for two human cell lines, HepG2 and 293 T, derived from different tissues (from liver for HepG2, from embryonic kidney for 293 T). We also re-tested the original P_SV40_::FLuc::3′-UTR reporter in 293 T cells (Figure 1d) and in U2OS cells (a human osteosarcoma cell line, Supplementary Figure S2), and found that FLuc was expressed at significantly higher levels when coupled to the 3111C-bearing 3′-UTR (*P<*0.01). However, when the P_SV40_::FLuc::3′-UTR reporter was expressed in the originally tested monkey Cos-1 cell line, there was no significant enhancement of FLuc expression by the SNP (Supplementary Figure S2), which agrees with the previous report.^33^ Therefore, although there is no difference between the *hClock* versus SV40 5′ regions (promoters) in their interaction with the 3′-UTR, the 3111C SNP significantly enhances the expression of the FLuc-3′-UTR (*hClock*) reporter in cell type/tissue-dependent manner. Taken together, the data indicate that in human cells the rs1801260 SNP enhances expression of the FLuc reporter and therefore strongly suggest that the rs1801260 SNP will modify expression of the hCLOCK protein, which is a key molecular component of the human circadian system.^17, 18, 19, 20^ Because the clock (via hCLOCK/ARNTL-mediated transcription) regulates expression of at least 15% of mammalian mRNAs in tissues throughout the body,^23, 24^ enhanced levels of hCLOCK are likely to modify these circadian output processes.

### Genetic association analyses in humans

This newly described functional impact of 3111C of *hClock* motivated us to re-evaluate its association with MDD. As compared with previous GWAS or meta-analyses,^4, 13, 14, 15, 35, 36^ the benefit of a more focused hypothesis-driven candidate gene study based on prior biological knowledge is the greatly reduced multiple testing burden, especially when coordinated biological information can be collected. We, therefore, undertook a candidate gene study that focused upon a small number of common polymorphisms (including rs1801260) in circadian clock genes to test an association with MDD, performing both combined and gender-stratified analyses, the latter based on the >50% higher prevalence of MDD in females. Our study sample included a subset of 1368 self-described Caucasian subjects derived from the depression (MDD) and control samples collected by the NIMH (USA) Center for Collaborative Genetic Studies.^25^ A diagnosis of subtype 1 MDD (melancholia) was used to define MDD case status; participants with no evidence of any form of MDD, either by clinical diagnosis or via targeted survey questions, were labeled study controls. After quality control procedures, 592 MDD cases and 776 MDD controls remained for analyses (further described in Supplementary Materials and Methods and demographic information in Supplementary Tables S1A and S1B). Samples were genotyped for a total of 32 genetic variants from eight circadian genes common in the Caucasian population^27^(Supplementary Table S2). These markers were chosen based on their common frequency in the Caucasian population and/or their potential functional significance based on well-characterized roles in regulating circadian rhythms and/or sleep disorders.^27, 28^

After quality control, 18 genetic variants remained for analysis in our data set (Supplementary Tables S3A and D). Genotypic and allelic chi-square (*χ*^2^) analyses (or where appropriate Fisher's exact tests) were performed in the full data set and in gender-stratified subsets of the data (see analysis flow chart, Supplementary Figure S3) to identify significant associations between genetic variants and susceptibility to MDD (Table 1, nonsignificant associations shown in Supplementary Table S4). Five variants had genotypic or allelic *χ*^2^ *P*-values less than 0.05 in the full and/or sex-stratified data sets (rs228697, rs17031614, rs4851377, rs34705978 and rs1801260). The variants with *P<*0.2 in the full data set were further assessed in gender-stratified subsets of the data to explore the genetic factors that may associate with MDD in males or females only. Of the three variants significant in the total data set (rs228697, rs17031614 and rs4851377), only rs228697 in *hPer3* associated in a gender-specific analysis (female *P*=0.041; Table 1). However, sex-stratified analyses identified two variants that were significantly associated with MDD in only one gender; the *hClock* SNP rs1801260 was associated with MDD in males (*P*=0.028), and rs34705978 in *hNPAS2* in females (*P*=0.034; Table 1). Of the two variants in *hPer3,* rs228697 (0.007<*P*<0.011) and rs17031614 (0.017<*P*<0.026), only the association with rs228697 remained significant after correction for multiple testing (Table 1). In addition, a single variant in *hNpas2*, rs4851377 (*P*=0.034) was associated with MDD susceptibility in the full data set, however significance did not remain after multiple testing correction.

### MDD associates with a *hClock* variant in males and a *hPer3* variant in females

Several studies, including a recent meta-analysis, failed to report an association between MDD and the rs1801260 SNP of *hClock*.^4, 13, 14, 15, 35, 36^ However, the majority of these studies were in Asian populations and did not include gender-stratified analyses. Because MDD is more common in females, it is highly likely that MDD data sets are heavily biased towards females and if rs1801260 were interrogated in such a data set, the association signal would likely have been masked, as it was in our combined male/female analysis (Table 1). In addition, if the effect of rs1801260 on susceptibility to MDD is also population specific, the lack of association in Asian populations may not be generalizable to other continental populations.^35^ Our discovery of a functional impact of this SNP on luciferase activity when expressed in human cells (Figure 1) encouraged us to re-evaluate previous association reports and include this marker in our association analyses, leading to the identification of a significant association by *χ*^2^ analysis between rs1801260 in *hClock* and MDD in male study participants (Table 1).

Furthermore, an independent analysis testing for association, prevalence-based association testing,^32^ provided additional evidence for the significant association with four variants previously identified using *χ*^2^ analyses (rs228697 and rs17031614 in hPER3, and rs485133 and rs34705978 in hNPAS2; Supplementary Table S5). Prevalence-based association testing analysis in the male subset revealed a novel association between MDD and a single variant in Arntl2, rs7137588 (*P*=0.008; Supplementary Table S5). Although none of the SNPs reached significance at the *P*⩽0.05 level in the male case-only data set in the prevalence-based association testing analysis, rs1801260 of *hClock* was the only SNP in the male case-only subset that was marginally significant (*P*=0.081, Supplementary Table S5). Finally, univariate logistic regression analyses using an additive model were performed to determine the strength and direction of effects for variants that were determined to be significantly associated with MDD in our data set (Table 2). In *hPer3*, rs228697 and rs17031614 were significantly associated with an increased risk for MDD in the full data set, while rs228697 was also significantly associated in the female-only subset. Moreover, rs1801260 in *hClock* was again significantly associated with a decreased risk for MDD in the male-only subset.

### *hClock* variant shows differential transcriptional response to glucocorticoids

On the basis of our results indicating a functional difference between the two alleles at 3111 in the hCLOCK 3′-UTR and the gender-specific association results, we tested whether hormones that might recapitulate the *in vivo* gender-specific environments affect expression *in vitro*. Neither testosterone nor estrogen influenced the expression of FLuc in the assay (not shown). However, the synthetic steroid glucocorticoid dexamethasone (Dex) had an unexpected effect on the expression assay (Figure 1e). In HepG2 cells that were transfected with the P_hClock_::FLuc::3′-UTR reporter, Dex from 50 to 500 nm significantly increased the expression level of FLuc relative to 0 nm Dex in the presence of the major allele (T), whereas constructs with the minor allele (C) had the opposite effect, namely Dex decreased the level of expression (*P<*0.001, Figure 1e). This result is interesting for three reasons. First, analysis of transcriptional factor binding motifs of the 3′ region of *hClock* revealed a glucocorticoid response element (GRE) in the SNP region (Supplementary Figure S4). Previous analyses of GRE-containing promoter sequences^37^ predict the T to C polymorphism at 3111 in *hClock* converting the putative GRE from a ‘repressed GRE motif' toward an ‘activated GRE motif' (Supplementary Figure S4). On the basis of the glucocorticoid responses of many genes,^37^ this conversion will reverse the glucocorticoid response. We also observed a reversal in the response to glucocorticoids for the rs1801260 variant in the 3′-UTR of the *hClock* gene (Figure 1e). Second, cortisol levels differ between females and males,^38, 39, 40^ and this difference correlates with the sex-dependent association of MDD with rs1801260 (Table 1). Finally, recent studies indicate that modulation of glucocorticoid-mediated stress response may constitute a common mechanism by which the circadian clock affects psychiatric disorders.^41^ Overall, the data suggest that sex differences in glucocorticoid levels may influence expression of *hClock* and thereby lead to sex-dependent effects on clock-influenced gene expression pathways, thereby altering susceptibility to MDD in a gender-specific manner.

### *hPer3* variant is a stronger transcriptional repressor

**Figure 2:**
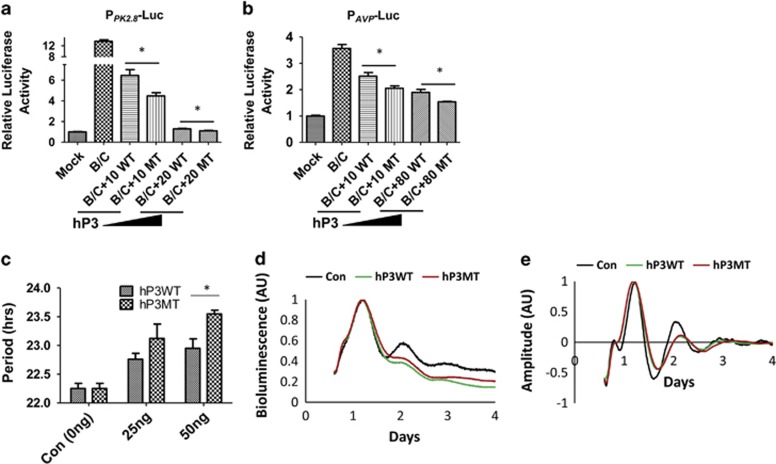
(**a** and **b**) The *hPer3* variant is a more potent transcriptional repressor of E-box *cis*-regulatory elements. Expression of hPER3 represses the BMAL1/CLOCK transactivation of two different E-box containing promoters (**a**, PK2.8; **b**, Avp) as assessed by a transient transfection assay in HEK 293 cells. Data are mean±s.e.m. (*n*=4) using firefly luciferase reporters (P_AVP_::FLuc and P_PK2.8_::FLuc) normalized by a *Renilla* luciferase control (P_CMV_::Rluc). Mock represents empty vectors; B/C represents coexpression of BMAL1/CLOCK; WT represents expression of native hPER3; MT represents expression of rs228697 SNP-containing hPER3 (as indicated, 10, 20, or 80  ng of hPer3 plasmid added to the transfection). *P<0.05, two-tailed unpaired *t*-test. (**c**) Period analyses of circadian rhythms in mammalian Rat-1 fibroblasts co-transfected with a P_*Bmal1*_::Luc reporter plasmid and a plasmid expressing either native hPER3 (hP3WT) or the hPER3 variant (hP3MT, rs228697 SNP-containing hPER3; mean±s.e.m., *n*=5 for 25 ng treatment, *n*=4 for 50 ng treatment, *n*=8 for 0 ng treatment). Two-way analysis of variance statistical analyses show significant differences for both genotype (WT versus MT, *P<*0.01) and for plasmid dosage (0, 25, 50 ng, *P*<0.001). *P<0.05, Bonferroni post tests. (**d**) Raw uncorrected data of Rat-1 fibroblasts that were co-transfected with 1 μg P_*Bmal1*_::Luc reporter plasmid and 50 ng pCDNA3 vector including either hP3WT or hP3MT, and pCDNA3.1 empty vector was used as control. The highest bioluminescence of each trace was set as 1. (**e**) Bioluminescence rhythms detrended from the raw data depicted in **d**. The highest detrended bioluminescence value of each trace was adjusted to 1. SNP, single-nucleotide polymorphism.

Coordination of functional assays was also examined for variants in *hPer3, hNpas2 and Arntl2* that showed significant association results (Tables 1 and 2, Supplementary Table S5). Appropriate functional tests are difficult to design for the variants rs17031614 (synonymous codon in *hPer3*), rs4851377 and rs34705978 (intron variants in *hNpas2*), and rs10548381 (upstream variant in *Arntl2*). However the rs228697 SNP is a missense mutation in the coding region of *hPer3* that changes a proline at position 856 to an alanine residue. Proline residues often have profound effects upon protein structure, so we tested the impact of this polymorphism in a transcriptional assay designed to test the regulation of E-box-containing promoters by CLOCK and BMAL1.^18, 19, 20^ PER3 is known to repress CLOCK/BMAL activation of E-box *cis*-reporters, and therefore we tested the repressive capability of hPER3 with and without the rs228697 SNP. As shown in Figures 2a and b, the variant-containing hPER3 is a stronger repressor of CLOCK/BMAL-stimulated transcription on two different E-box *cis*-reporters (P_PK2.8_ and P_AVP_). As compared with the protein encoded by the common allele, the rs228697 variant stabilizes hPER3 (Supplementary Figure S5) and recruits more PER2 into a transcription repression complex (Supplementary Figure S6). Because PER3, PER2 and PER1 interact to synergistically promote nuclear translocation,^20^ the rs228697 variant augmentation of hPER3 stability and PER complex formation predict enhanced repressive activity, as depicted in Figures 2a and b. Moreover, when hPER3 is transiently expressed in mammalian fibroblasts, the rs228697 variant causes a significant dose-dependent lengthening of the circadian period as compared with the native version of hPer3 (Figures 2c–e). Although this period effect is statistically significant, the small magnitude of the period effect indicates that the primary impact of the rs228697 hPer3 variant is likely to be upon clock-regulated transcription pathways (as in Figures 2a and b) rather than upon the central clock mechanism itself.

## Discussion

### Molecular assays of functional activity affected by *hClock* and *hPer3* polymorphisms

Functional and association analyses indicate that common polymorphisms in core circadian genes affect the activity of the encoded proteins and provide a connection between clock function and MDD. Critical is the sex-dependent nature of these associations with MDD, which is a sexually dimorphic disorder.^41^ The rs1801260 allele is protective in males, so males with this polymorphism are less likely to present with MDD. The rs1801260 polymorphism in the 3′-untranslated region (3′-UTR) of the *hClock* gene^33^ stabilizes a reporter construct when expressed in the human cell lines (Figure 1), and higher abundance of the hCLOCK protein is known to alter circadian period and phase.^17^ Glucocorticoid levels exhibit age-dependent differences between males and females.^38, 39^ Moreover, 3′ noncoding regions of other transcripts contain glucocorticoid-responsive elements that regulate activity.^42^ As glucocorticoids reciprocally modulated the expression of the 3′-UTR reporter *in vitro* in an allele-specific manner (Figure 1e), our results revealed a potential glucocorticoid regulatory region in the 3′-UTR of the *hClock* transcript, which resonates with recent proposals suggesting that glucocorticoid-mediated stress response constitutes a common mechanism by which the circadian clock affects psychiatric disorders including MDD.^41, 43^ Finally, the 3′-UTR of the mouse homolog of *Clock* is significantly different from that of *hClock*, and, in particular, *mClock* does not include a homologous region to that of the rs1801260 region of *hClock*. Consequently, although *mClock*-mutant mice have been proposed as models of mania,^44^ they will not allow an adequate test of a human stress/MDD connection mediated through the rs1801260 region of the *hClock* 3′-UTR.

The re-analyses of association of circadian gene polymorphisms with MDD also revealed potential associations within *hPer3* and *hNpas2* (Table 1, Supplementary Table S5). As in the case with rs1801260, these associations were sex-dependent, but unlike *hClock*'s rs1801260, SNPs within *hPer3* (rs228697) and *hNpas2* (rs34705978) strongly associated with MDD in females (Table 1). The NPAS2/ARNTL1 complex is a key regulator of circadian transcriptional activation;^17^ the rs4851377 and rs34705978 SNPs of *hNpas2* are both intronic and might influence expression of the NPAS2. The PER3 protein is part of the negative feedback process that controls circadian transcriptional networks and its effects are highly tissue dependent; knockout of *Per3* in mice does not cause significant effects on the central clock mechanism in the brain,^45^ but it has large effects on clock-regulated gene expression in other tissues such as liver, colon, esophagus and adipose tissue.^46^ Although the hPer3 variant that we studied here has a significant influence on circadian period (Figures 2c–e), this effect is smaller than its action on PER-modulated gene expression (Figures 2a/b), suggesting that rs228697 hPer3 variant acts more strongly on clock-regulated output pathways than upon the central clockwork. Moreover, expression of *Per3* may be sex dependent, as interactions exist between the estrogen receptor, menstrual/estrus cycles and expression of *Per* genes in humans and rodents.^16, 47^ The exonic SNP rs228697 is predicted to change a proline to an alanine within the hPER3 amino acid sequence, and our functional data support the conclusion that the rs228697 variant slows hPER3 turnover, thereby enhancing the effective repressive activity of hPER3 on CLOCK/BMAL1-mediated transcriptional activity (Figures 2a and b, Supplementary Figures S5 and S6). Interestingly, rs228697 of hPer3 is significantly associated with altered phasing of daily activity/sleep timing,^48^ which together with our observations (Tables 1 and 2, Supplementary Table S5 and Figure 2) resurrects a hypothesis that MDD is at least partially due to suboptimal phasing of activity/sleep with the environment.^2, 3, 4, 6^ If true, circadian phase resetting may be an effective therapy. Intriguingly, our results with rs1801260 of *hClock* are also consistent with a phasing interpretation because a stabilization of hCLOCK that leads to increased protein levels should alter circadian period and phase.^17^ Indeed, the first report of rs1801260 of *hClock* suggested its impact upon human phase,^34^ and therefore rs1801260 of *hClock* and rs228697 of hPer3 are similar in sharing a delayed phase phenotype (specifically, a delayed morningness–eveningness preference).^34, 48^

### Sex-dependent association of clock gene polymorphisms with MDD

Studies of the affective disorders MDD, bipolar disorder and seasonal affective disorder have long suggested that disrupted circadian (daily) timing mechanisms may contribute to the development or severity of depression,^1, 2, 3, 4, 6, 7^ but genetic links between clocks and mood disorders have been tenuous.^4, 11, 12, 14, 15, 35, 36, 49^ However, the earlier case/control attempts to associate rs1801260 in *hClock* used sample sizes that were relatively small and did not stratify for sex.^35, 36^ Given that MDD is much more common in females, failure to stratify may have led to an association of rs1801260 with MDD being overlooked in males, especially as the effect of this SNP in females was so close to unity (odds ratio=0.95). Moreover, meta-analyses that combined different ethnic groups to increase sample size,^35^ could mask associations that are population specific. Previous GWAS and/or meta-analyses that used large sample sizes and which searched for polymorphisms that associate with MDD did not discover variants in clock genes.^13, 14, 15^ But those studies may have suffered from inconsistent phenotype criteria and the enhanced multiple testing burden that is incumbent in tests of thousands of SNPs and multiple phenotypes. Also these studies did not perform gender-stratified analyses. We believe that our study revealed heretofore unappreciated associations because of a combination of factors: (1) a hypothesis-driven approach inspired and enhanced by functional data, (2) a targeted case/control analysis of a limited number of polymorphisms and phenotypes, (3) a consistent set of criteria that were professionally assessed by the NIMH Center for Collaborative Genetic Studies project and (4) a sex-stratified approach enabled by a sample size with a sufficient number of males to make gender comparisons. A further key is that we connected association results to explicit functional differences at the cell level.

We propose that the impact of the *hClock* and *hPer3* SNPs on transcription and/or expression (Figures 1 and 2) may not be on the core circadian oscillator, but on global output transcriptional pathways^17, 23, 24^ that are mediated sex-dependently by the circadian system. This interpretation is consistent with (and potentially explanatory for) observations of disrupted transcriptional patterns in the brains of humans suffering from MDD.^8^ These output pathways can include targets of CLOCK-mediated E-box transcription such as the neuropeptide cholecystokinin that is involved in mood disorders.^50^ Moreover, this clock system controls neurotransmitters and their receptors (for example, serotonin) that are known to be modulators of mood.^4^ As such, the polymorphisms that we report herein reveal core circadian genes to be targeted for functional and clinical studies to understand the connections between the circadian system and MDD, which may ultimately lead to noninvasive therapies that ameliorate the symptoms of MDD.^51^

## Figures and Tables

**Table 1:** Genetic loci significantly associated with MDD in combined data set and/or sex-separated subsets

*Chr*	*SNP*	*Gene*	*Test*^a^	*Combined* P-*value*^b^	*Female* P-*value*^c^	*Male* P-*value*^d^
1	rs228697	PER3	Geno	**0.011**	0.055	0.440^e^
1	rs228697	PER3	Allelic	**0.007**^f^	**0.041**	0.255
						
1	rs17031614	PER3	Geno	**0.026**^e^	0.119^e^	0.108^e^
1	rs17031614	PER3	Allelic	**0.017**	0.076	0.135
						
2	rs4851377	NPAS2	Geno	**0.034**	0.081	0.748
2	rs4851377	NPAS2	Allelic	0.070	0.125	0.839
						
2	rs34705978	NPAS2	Geno	0.056	**0.034**	0.974^e^
2	rs34705978	NPAS2	Allelic	0.184	0.121	0.679
						
4	rs1801260	CLOCK	Geno	0.402	0.883	0.079
4	rs1801260	CLOCK	Allelic	0.178	0.622	**0.028**
						
11	rs70965440	ARNTL	Geno	0.152^e^	0.076^e^	0.462^e^
11	rs70965440	ARNTL	Allelic	0.237	0.111	0.611

Abbreviations: MDD, major depressive disorder; SNP, single-nucleotide polymorphism.

a Describes the type of chi-square test performed; genotypic (Geno) or allelic.

b *P*-values are from chi-square test performed in the combined data set unless otherwise noted.

c *P*-values are from chi-square tests performed in the female-only subset unless otherwise noted.

d *P*-values are from chi-square tests performed in the male-only subset unless otherwise noted.

e Fisher's exact *P*-value or one-sided Fisher's exact *P*-value is shown.

f SNP remained significant after multiple testing correction with false discovery rate *q*=0.2.

Significant *P*-values (*P*⩽0.05) are shown in bold. Gender-separated studies performed only for select SNPs.

**Table 2:** Logistic regression analysis of significant loci from chi-square or PRAT analyses

*Chr*	*SNP*	*Gene*	*Combined odds ratio*	*Combined* P-*value*	*Female odds ratio*	*Female* P-*value*	*Male odds ratio*	*Male* P-*value*
1	rs228697	PER3	1.39	**0.007**	1.38	**0.041**	1.38	0.261
1	rs17031614	PER3	1.68^a^	**0.022**^a^	1.52^b^	0.091^b^	2.04	0.217
2	rs4851377	NPAS2	0.87	0.073	0.88	0.136	0.97	0.843
2	rs34705978	NPAS2	1.14	0.186	1.19	0.116	0.91	0.684
4	rs1801260	CLOCK	0.89	0.187	0.96	0.647	0.66	**0.036**
12	rs10548381	ARNTL2	1.04	0.759	0.96	0.762	1.37	0.245

Abbreviations: PRAT, prevalence-based association testing; SNP, single-nucleotide polymorphism.

a Indicates that exact logistic regression analysis was used.

b Exact logistic regression was attempted but was not computationally feasible, therefore standard logistic regression analysis using a dominant genetic model was applied.

An odds ratio >1 indicates that possessing the minor allele at this variant increases the odds of MDD; an odds ratio <1 indicates that possessing the minor allele at this variant decreases the odds of MDD. Significant *P*-values (*P*⩽0.05) are shown in bold.

## References

[bib1] Giedke H, Schwärzler F. Therapeutic use of sleep deprivation in depression. Sleep Med Rev 2002; 6: 361–377. 12531127

[bib2] Levandovski R, Dantas G, Fernandes LC, Caumo W, Torres I, Roenneberg T et al. Depression scores associate with chronotype and social jetlag in a rural population. Chronobiol Int 2011; 28: 771–778. 21895489 10.3109/07420528.2011.602445

[bib3] Lewy AJ. Circadian misalignment in mood disturbances. Curr Psychiatry Rep 2009; 11: 459–465. 19909668 10.1007/s11920-009-0070-5

[bib4] McClung CA. Circadian genes, rhythms and the biology of mood disorders. Pharmacol Ther 2007; 114: 222–232. 17395264 10.1016/j.pharmthera.2007.02.003 PMC1925042

[bib5] Wehr TA, Goodwin FK. Circadian Rhythms in Psychiatry. Boxwood Press: Pacific Grove, CA, USA, 1983.

[bib6] Wehr TA, Wirz-Justice A, Goodwin FK, Duncan W, Gillin JC. Phase advance of the circadian sleep-wake cycle as an antidepressant. Science 1979; 206: 710–713. 227056 10.1126/science.227056

[bib7] Lewy AJ, Sack RL, Miller LS, Hoban TM. Antidepressant and circadian phase-shifting effects of light. Science 1987; 235: 352–354. 3798117 10.1126/science.3798117

[bib8] Li JZ, Bunney BG, Meng F, Hagenauer MH, Walsh DM, Vawter MP et al. Circadian patterns of gene expression in the human brain and disruption in major depressive disorder. Proc Natl Acad Sci USA 2013; 110: 9950–9955. 23671070 10.1073/pnas.1305814110 PMC3683716

[bib9] Kelsoe JR. Arguments for the genetic basis of the bipolar spectrum. J Affect Disord 2003; 73: 183–197. 12507751 10.1016/s0165-0327(02)00323-3

[bib10] Sullivan PF, Neale MC, Kendler KS. Genetic epidemiology of major depression: review and meta-analysis. Am J Psychiatry 2000; 157: 1552–1562. 11007705 10.1176/appi.ajp.157.10.1552

[bib11] Benedetti F, Serretti A, Colombo C, Barbini B, Lorenzi C, Campori E et al. Influence of CLOCK gene polymorphism on circadian mood fluctuation and illness recurrence in bipolar depression. Am J Med Genet B Neuropsychiatr Genet 2003; 123: 23–26. 10.1002/ajmg.b.20038 14582141

[bib12] Kennaway DJ. Clock genes at the heart of depression. J Psychopharmacol 2010; 24: 5–14. 10.1177/1359786810372980 20663803

[bib13] McCarthy MJ, Nievergelt CM, Kelsoe JR, Welsh DK. A survey of genomic studies supports association of circadian clock genes with bipolar disorder spectrum illnesses and lithium response. PLoS One 2012; 7: e32091. 22384149 10.1371/journal.pone.0032091 PMC3285204

[bib14] Byrne EM, Heath AC, Madden PAF, Pergadia ML, Hickie IB, Montgomery GW et al. Testing the role of circadian genes in conferring risk for psychiatric disorders. Am J Med Genet B Neuropsychiatr Genet 2014; 165B: 254–260. 24687905 10.1002/ajmg.b.32230 PMC4397914

[bib15] Hek K, Demirkan A, Lahti J, Terracciano A, Teumer A, Cornelis MC et al. A genome-wide association study of depressive symptoms. Biol Psychiatry 2013; 73: 667–678. 23290196 10.1016/j.biopsych.2012.09.033 PMC3845085

[bib16] Bailey M, Silver R. Sex differences in circadian timing systems: implications for disease. Front Neuroendocrinol 2014; 35: 111–139. 24287074 10.1016/j.yfrne.2013.11.003 PMC4041593

[bib17] Lowrey PL, Takahashi JS. Genetics of circadian rhythms in mammalian model organisms. Adv Genet 2011; 74: 175–230. 21924978 10.1016/B978-0-12-387690-4.00006-4 PMC3709251

[bib18] Gekakis N, Staknis D, Nguyen HB, Davis FC, Wilsbacher LD, King DP et al. Role of the CLOCK protein in the mammalian circadian mechanism. Science 1998; 280: 1564–1569. 9616112 10.1126/science.280.5369.1564

[bib19] Zylka M, Shearman L, Weaver D, Reppert SM. Three period homologs in mammals: differential light responses in the suprachiasmatic circadian clock and oscillating transcripts outside the brain. Neuron 1998; 20: 1103–1110. 9655499 10.1016/s0896-6273(00)80492-4

[bib20] Kume K, Zylka MJ, Sriram S, Shearman LP, Weaver DR, Jin X et al. mCRY1 and mCRY2 are essential components of the negative limb of the circadian clock feedback loop. Cell 1999; 98: 193–205. 10428031 10.1016/s0092-8674(00)81014-4

[bib21] DeBruyne JP, Weaver DR, Reppert SM. CLOCK and NPAS2 have overlapping roles in the suprachiasmatic circadian clock. Nat Neurosci 2007; 10: 543–545. 17417633 10.1038/nn1884 PMC2782643

[bib22] Reick M, Garcia JA, Dudley C, McKnight SL. NPAS2: an analog of Clock operative in the mammalian forebrain. Science 2001; 293: 506–509. 11441147 10.1126/science.1060699

[bib23] Panda S, Antoch MP, Miller BH, Su AI, Schook AB, Straume M et al. Coordinated transcription of key pathways in the mouse by the circadian clock. Cell 2002; 109: 307–320. 12015981 10.1016/s0092-8674(02)00722-5

[bib24] Miller BH, McDearmon EL, Panda S, Hayes K, Zhang J, Andrews JL et al. Circadian and CLOCK-controlled regulation of the mouse transcriptome and cell proliferation. Proc Natl Acad Sci USA 2007; 104: 3342–3347. 17360649 10.1073/pnas.0611724104 PMC1802006

[bib25] Levinson DF, Zubenko GS, Crowe RR, DePaulo RJ, Scheftner WS, Weissman MM et al. Genetics of recurrent early-onset depression (GenRED): design and preliminary clinical characteristics of a repository sample for genetic linkage studies. Am J Med Genet B Neuropsychiatr Genet 2003; 119: 118–130. 10.1002/ajmg.b.20009 12707949

[bib26] Kessler RC, Andrews G, Mroczek D, Üstün TB, Wittchen HU. The World Health Organization Composite International Diagnostic Interview Short Form (CIDI-SF). Int J Methods Psychiatr Res 1998; 7: 171–185.

[bib27] Ciarleglio CM, Ryckman k, Servick SV, Hida A, Robbins S, Wells N et al. Genetic differences in human circadian clock genes among worldwide populations. J Biol Rhythms 2008; 23: 330–340. 18663240 10.1177/0748730408320284 PMC2579796

[bib28] Gamble KL, Motsinger-Reif AA, Hida A, Borsetti HM, Servick SV, Ciarleglio CM et al. Shift work in nurses: contribution of phenotypes and genotypes to adaptation. PLoS One 2011; 6: e18395. 21533241 10.1371/journal.pone.0018395 PMC3076422

[bib29] Purcell S, Neale B, Todd-Brown K, Thomas L, Ferreira MA, Bender D et al. PLINK: a tool set for whole-genome association and population-based linkage analyses. Am J Hum Genet 2007; 81: 559–575. 17701901 10.1086/519795 PMC1950838

[bib30] Dupont WD, Plummer WD. PS power and sample size program available for free on the Internet. Control Clin Trials 1997; 18: 274.

[bib31] Benjamini N, Hochberg Y. Controlling the false discovery rate: a practical and powerful approach to multiple testing. J R Stat Soc Ser B 1995; 57: 289–300.

[bib32] Ryckman KK, Jiang L, Li C, Bartlett J, Haines JL, Williams SM. A prevalence-based association test for case-control studies. Genet Epidemiol 2008; 32: 600–605. 18473366 10.1002/gepi.20342

[bib33] Robilliard DL, Archer SN, Arendt J, Lockley SW, Hack LM, English J et al. The 3111 Clock gene polymorphism is not associated with sleep and circadian rhythmicity in phenotypically characterized human subjects. J Sleep Res 2002; 11: 305–312. 12464098 10.1046/j.1365-2869.2002.00320.x

[bib34] Katzenberg D, Young T, Finn L, Lin L, King DP, Takahashi JS et al. A CLOCK polymorphism associated with human diurnal preference. Sleep 1998; 21: 569–576. 9779516 10.1093/sleep/21.6.569

[bib35] Kishi T, Yoshimura R, Fukuo Y, Kitajima T, Okochi T, Matsunaga S et al. The CLOCK gene and mood disorders: a case-control study and meta-analysis. Chronobiol Int 2011; 28: 825–833. 22080789 10.3109/07420528.2011.609951

[bib36] Desan PH, Oren DA, Malison R, Price LH, Rosenbaum J, Smoller J et al. Genetic polymorphism at the CLOCK gene locus and major depression. Am J Med Genet 2000; 96: 418–421. 10898925 10.1002/1096-8628(20000612)96:3<418::aid-ajmg34>3.0.co;2-s

[bib37] Kuo T, Lew MJ, Mayba O, Harris CA, Speed TP, Wang JC. Genome-wide analysis of glucocorticoid receptor-binding sites in myotubes identifies gene networks modulating insulin signaling. Proc Natl Acad Sci USA 2012; 109: 11160–11165. 22733784 10.1073/pnas.1111334109 PMC3396543

[bib38] Halbreich U, Asnis GM, Zumoff B, Nathan RS, Shindledecker R. Effect of age and sex on cortisol secretion in depressives and normals. Psychiatry Res 1984; 13: 221–229. 6597461 10.1016/0165-1781(84)90037-4

[bib39] Halbreich U, Lumley LA. The multiple interactional biological processes that might lead to depression and gender differences in its appearance. J Affect Disord 1993; 29: 159–173. 7905489 10.1016/0165-0327(93)90030-n

[bib40] Halbreich U, Ray O. Hormones, brain, and neuropsychopharmacology. Neuropsychopharmacology 2000; 23: VIII. 10891644 10.1016/S0893-133X(00)00128-7

[bib41] Landgraf D, McCarthy MJ, Welsh DK. Circadian clock and stress interactions in the molecular biology of psychiatric disorders. Curr Psychiatry Rep 2014; 16: 483. 25135782 10.1007/s11920-014-0483-7

[bib42] Petersen DD, Koch SR, Granner DK. 3' noncoding region of phosphoenolpyruvate carboxykinase mRNA contains a glucocorticoid-responsive mRNA-stabilizing element. Proc Natl Acad Sci USA 1989; 86: 7800–7804. 2813358 10.1073/pnas.86.20.7800 PMC298158

[bib43] Liliavski A, Dumbell R, Ott V, Oster H. Adrenal clocks and the role of adrenal hormones in the regulation of circadian physiology. J Biol Rhythms 2015; 30: 20–34. 25367898 10.1177/0748730414553971

[bib44] Roybal K, Theobold D, Graham A, DiNieri JA, Russo SJ, Krishnan V et al. Mania-like behavior induced by disruption of CLOCK. Proc Natl Acad Sci USA 2007; 104: 6406–6411. 17379666 10.1073/pnas.0609625104 PMC1851061

[bib45] Bae K, Jin X, Maywood ES, Hastings MH, Reppert SM, Weaver DR. Differential functions of mPer1, mPer2, and mPer3 in the SCN circadian clock. Neuron 2001; 30: 525–536. 11395012 10.1016/s0896-6273(01)00302-6

[bib46] Pendergast JS, Niswender KD, Yamazaki S. Tissue-specific function of Period3 in circadian rhythmicity. PLoS One 2012; 7: e30254. 22253927 10.1371/journal.pone.0030254 PMC3256228

[bib47] Nakamura TJ, Moriya T, Inoue S, Shimazoe T, Watanabe S, Ebihara S et al. Estrogen differentially regulates expression of Per1 and Per2 genes between central and peripheral clocks and between reproductive and nonreproductive tissues in female rats. J Neurosci Res 2005; 82: 622–630. 16273538 10.1002/jnr.20677

[bib48] Hida A, Kitamura S, Katayose Y, Kato M, Ono H, Kadotani H et al. Screening of clock gene polymorphisms demonstrates association of a PER3 polymorphism with morningness-eveningness preference and circadian rhythm sleep disorder. Sci Rep 2014; 4: 6309. 25201053 10.1038/srep06309 PMC4158573

[bib49] Soria V, Martínez-Amorós E, Escaramís G, Valero J, Pérez-Egea R, García C et al. Differential association of circadian genes with mood disorders: CRY1 and NPAS2 are associated with unipolar major depression and CLOCK and VIP with bipolar disorder. Neuropsychopharmacology 2010; 35: 1279–1289. 20072116 10.1038/npp.2009.230 PMC3055337

[bib50] Arey RN, Enwright JF 3rd, Spencer SM, Falcon E, Ozburn AR, Ghose S et al. An important role for cholecystokinin, a CLOCK target gene, in the development and treatment of manic-like behaviors. Mol Psychiatry 2014; 19: 342–350. 23399917 10.1038/mp.2013.12 PMC3783638

[bib51] Wirz-Justice A, Benedetti F, Terman M. Chronotherapeutics for Affective Disorders: A Clinician's Manual for Light and Wake Therapy. Karger Press: Basel, Switzerland, 2013.

