# Supplementary material for: Molecular analyses of circadian gene variants reveal sex-dependent links between depression and clocks
Source: Transl Psychiatry. 2016 Mar 1;6(3):e748–. doi: 10.1038/tp.2016.9 (PMC4872462; doi:10.1038/tp.2016.9)
Supplement: Supplementary Information [file tp20169x1.pdf]

Supplemental Information for Shi et al.:

Molecular Analyses of Circadian Gene Variants Reveal Sex-dependent Links Between Depression and Clocks

Supplemental Figures:

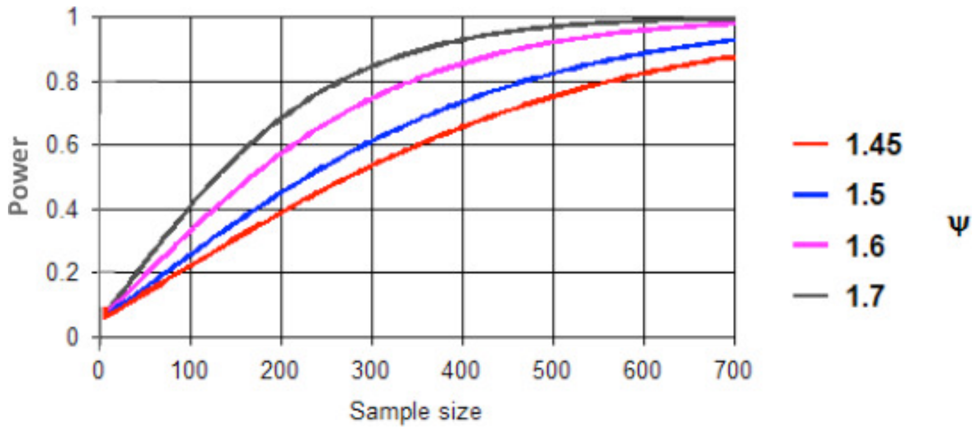

Figure S1, A priori power calculations for various effect sizes. Sample size refers to number of cases. Effect size is measured as the odds ratio from a logistic regression analysis of the relationship between genotype at a SNP with minor allele frequency 0.2 and susceptibility to MDD. Estimated power for various effect sizes is indicated by colored lines.

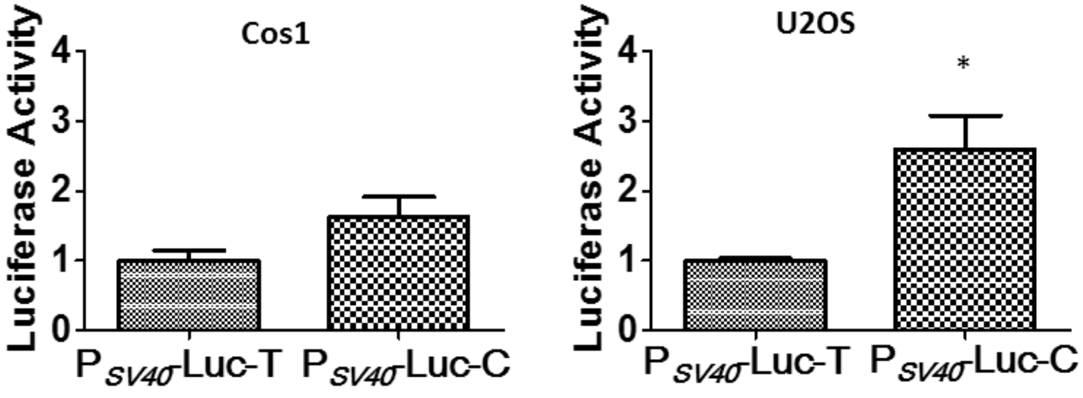

Figure S2, related to Figure 1. Functional analysis of *hClock* SNP in cell cultures.

(A) Cos-1 (monkey kidney) cells were transfected with P_{SV40}::FLuc::3' UTR.
(B) U2OS (human osteosarcoma) were transfected with P_{SV40}::FLuc::3' UTR.

Methods are as in Figure 1. Levels of firefly luciferase activity were normalized with the *Renilla* luciferase reporter (pCMV-Rluc). The activity in samples transfected with the reporter constructs containing the common 3'UTR allele (pCI-Luc-T or pSV40-luc-T) was set as 1.0. Results are shown as mean±SEM of two independent experiments (left panel, total n = 12; right panel, total n = 8). *$p < 0.05$ by two-tail unpaired t-test. The P_{SV40}::FLuc::3'UTR reporter was kindly provided by Dr. Malcolm von Schantz.

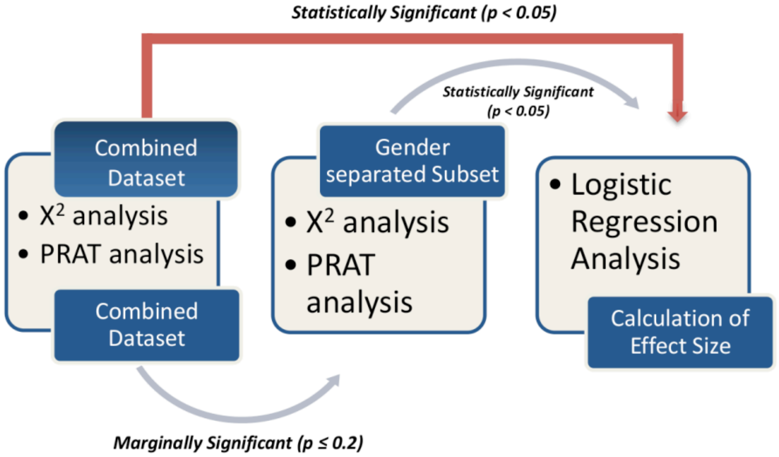

Figure S3, related to Tables 1 and 2. Statistical Analysis Flow Chart.

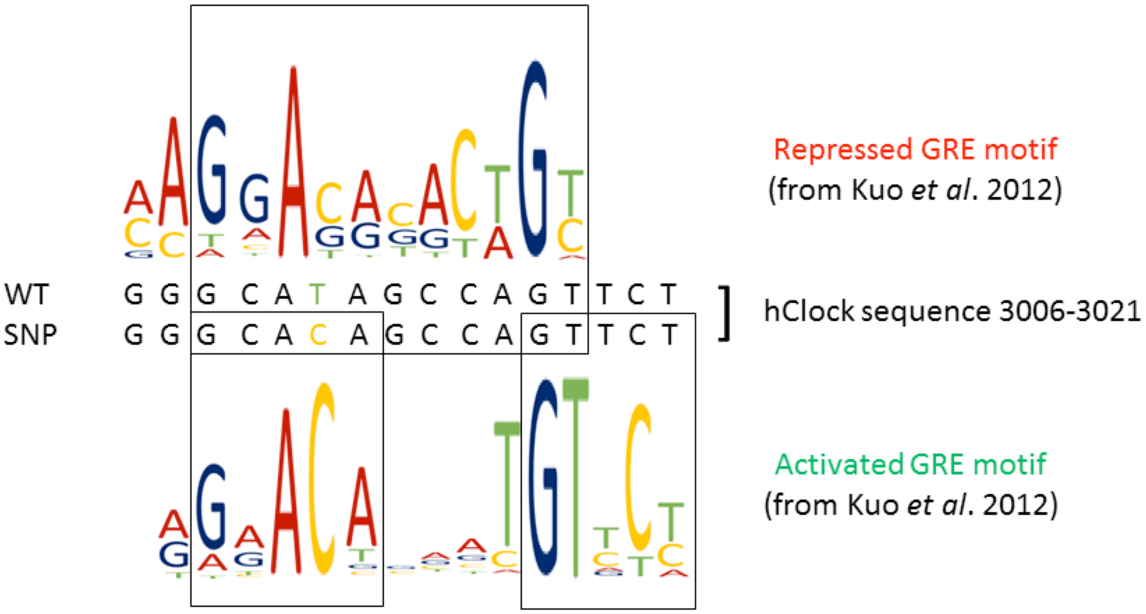

Figure S4, related to Figure 1. *hClock* expression is modulated by dexamethasone and the rs1801260 SNP. Schematic shows a glucocorticoid response element (GRE) in the SNP region of 3' UTR of the *hClock* gene. In this figure, the minor allele (rs1801260 SNP) is “C” (indicated by yellow font) and the major allele is “T” (indicated by green font). The consensus motifs of activated vs. repressed GREs are based on the analyses of Kuo and coworkers (1), and these portions of the figure are modified from that publication.

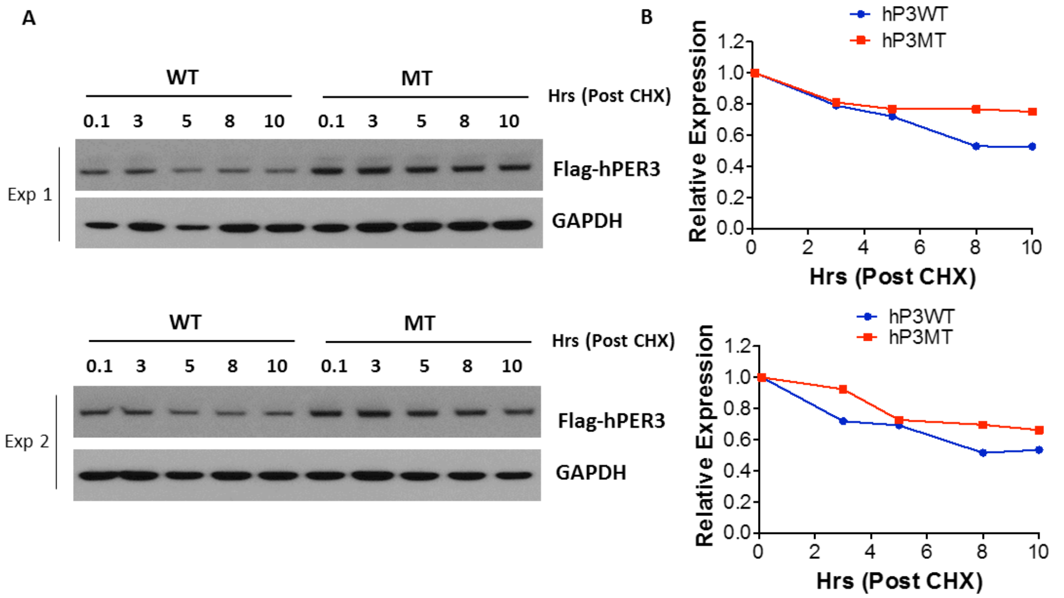

Figure S5, related to Figure 2. Degradation of hPER3 protein is slowed by the rs228697 variant. Immunoblotting was used to assess the transfected expression of Flag-hPER3 in HEK 293 after CHX treatment (GAPDH served as loading control, WT = wild-type, MT = rs228697 variant). Two independent immunoblots are shown in panel **A**, and quantification of Flag-hP3 protein expression normalized with GAPDH for each experiment is shown in panel **B** (for both Flag-hPer3-WT and Flag-hPer3-MT, the expression at the first time-point {0.1h} after CHX treatment was set as 1).

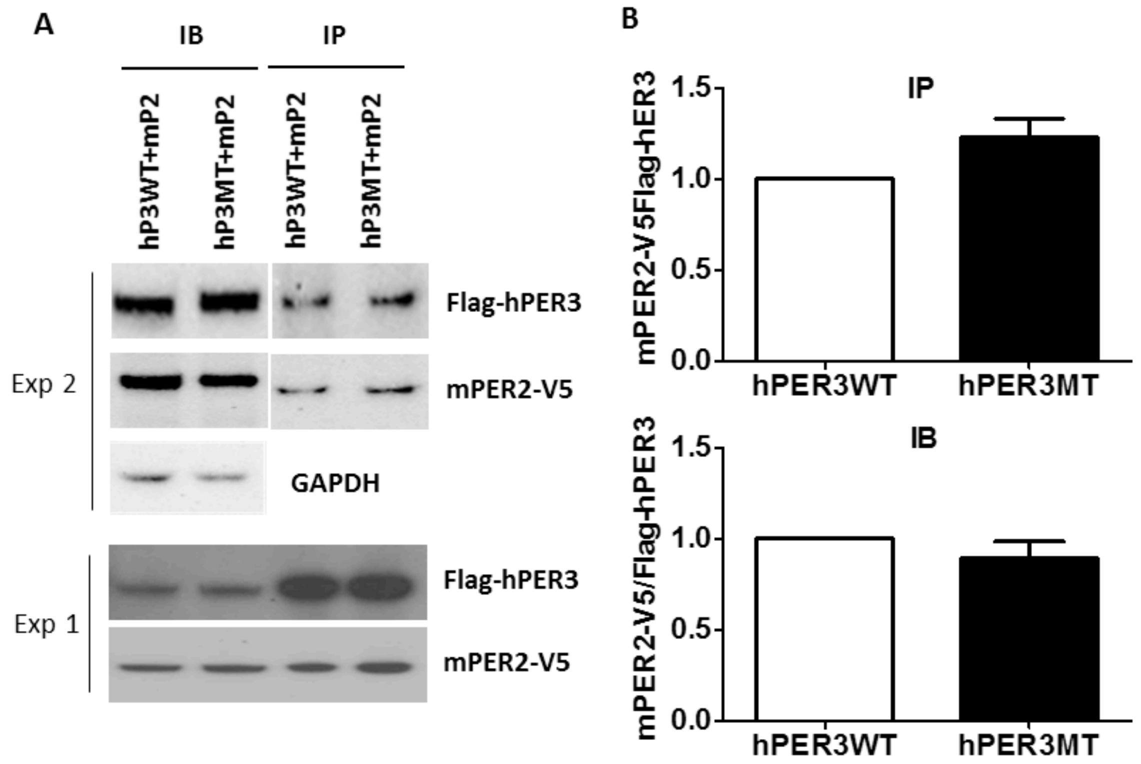

Fig S6, related to Figure 2. Interaction between PER2 and PER3 in HEK293 cells. HEK293 cells were co-transfected with Flag-h*Per3* with or without the rs228697 SNP (wildtype, WT; or rs228697 variant/mutation, MT) and m*Per2*-V5 (mP2). Immunoprecipitates were prepared using a limiting amount of anti-Flag antibody and the immunoprecipitates (IP) and original cell lysates (IB) were separated by electrophoresis and immunoblotted with either anti-Flag (for hPER3) or anti-V5 (for mPER2). **A**. Two independent immunoblot experiments (Exp 1/2). The input level of the cell lysates and of the anti-Flag antibody for IP in experiment 1 were approximately 3X more than those in experiment 2. GAPDH was served as loading control. **B**. Upper panel (IP): Quantification of the mPER2-V5 protein that was co-immunoprecipitated with Flag-hPER3 antibody. The amount of hPER3WT pull-down was normalized to 1.0. Lower panel (IB): Amount of hPER3WT (native hPER3) and hPER3MT (rs228697 variant/mutation) present in the cell lysates.

Table S1A. Demographic description of MDD Study Participants

Categorical Variable	Total	MDD Case	MDD Control	PVALUE
Study Participants	1368	592	776	
Age, yr[1,2]		40.92(10.83)	40.67(11.26)	0.488
Alcohol Abuse[3] (% of each category)		15.93%	15.52%	0.837
Drug Abuse[3] (% of each category)		7.77%	8.41%	0.669

[1] Variable is not normally distributed (Shapiro-Wilk test P-value < 0.05). P-value presented was determined using the Mann-Whitney U test.
[2] Mean (SD) for categorical variable
[3] P-value presented was calculated using standard Two -Sample Test of Proportions (Z Test)

Table S1B. Demographic description of MDD Study Participants by gender

Categorical Variable	Total	MDD Case	MDD Control	PVALUE
Female	1021	498	523	
Age, yr[1,2]		40.94(10.81)	41.20(11.14)	0.859
Alcohol Abuse[3] (% of each category)		14.31%	11.88%	0.221
Drug Abuse[3] (% of each category)		6.83%	7.69%	0.595
Male	347	94	253	
Age, yr[1,2]		40.83(10.99.)	39.56(11.45)	0.274
Alcohol Abuse[3] (% of each category)		24.47%	23.32%	0.823
Drug Abuse[3] (% of each category)		12.77%	9.88%	0.439

[1] Variable is not normally distributed (Shapiro-Wilk test P-value < 0.05). P-value presented was determined using the Mann-Whitney U test.
[2] Mean (SD) for categorical variable
[3] Pvalue presented was calculated using standard Two -Sample Test of Proportions (Z Test)

Table S2. Summary of all Genotyped Markers

CHR	SNP	Allele1	Allele2	Overall Allele1 frequency	Overall Allele2 frequency	Case Allele 1 frequency	Case Allele2 Frequency	Control Allele1 Frequency	Control Allele2 Frequency
1	AB047536	5	4	0.350	0.650	0.348	0.652	0.351	0.649
1	rs228669	A	G	0.076	0.924	0.083	0.917	0.071	0.929
1	rs35733104[1]	C	-	1.000	0.000	1.000	0.000	1.000	0.000
1	rs35899625[1]	G	T	$3.652e^{-4}$	1.000	0.001	0.999	0.000	1.000
1	rs228697	G	C	0.109	0.891	0.125	0.875	0.097	0.903
1	rs17031614	A	G	0.034	0.966	0.042	0.959	0.028	0.972
2	rs1811399	G	T	0.235	0.765	0.224	0.776	0.243	0.757
2	rs2117714	C	T	0.297	0.703	0.285	0.715	0.306	0.694
2	rs4851377	C	T	0.462	0.538	0.440	0.560	0.479	0.521
2	rs34705978	T	C	0.189	0.811	0.201	0.799	0.180	0.820
2	rs17717414	G	A	0.189	0.811	0.181	0.819	0.195	0.805
2	rs70965449[1]	G	-	1.000	0.000	1.000	0.000	1.000	0.000
2	rs70965448[1]	T	C	0.001	0.999	0.003	0.998	0.001	0.999
2	rs2304669	G	A	0.144	0.856	0.141	0.859	0.146	0.854
2	rs70965447[1]	G	-	1.000	0.000	1.000	0.000	1.000	0.000
4	rs70965446[1]	G	A	$3.546e^{-4}$	1.000	0.000	1.000	0.001	0.999
4	rs1801260	C	T	0.274	0.727	0.261	0.739	0.283	0.717
4	rs8192551[2]	G	A	0.042	0.958	0.035	0.965	0.047	0.953
4	rs8192550	G	A	0.039	0.961	0.034	0.966	0.043	0.957
11	rs70965440	G	T	0.015	0.985	0.018	0.982	0.012	0.988
11	rs70965441	C	A	0.028	0.973	0.027	0.973	0.028	0.972
11	rs70965442[1]	A	G	0.003	0.997	0.004	0.996	0.003	0.998
11	rs10830963	G	C	0.255	0.745	0.261	0.739	0.250	0.750
11	rs1562444	A	G	0.499	0.501	0.508	0.493	0.493	0.507
11	rs12792653[2]	A	G	0.499	0.501	0.508	0.493	0.493	0.507
12	rs70965443[1]	A	-	1.000	0.000	1.000	0.000	1.000	0.000
12	rs10548381	T	A	0.099	0.901	0.101	0.899	0.098	0.903
12	rs70965445[1]	A	-	1.000	0.000	1.000	0.000	1.000	0.000
12	rs7137588	C	G	0.362	0.638	0.367	0.633	0.359	0.641
12	rs4964059[3]	C	A	0.362	0.638	0.366	0.634	0.358	0.642
12	rs70965444[1]	T	-	1.000	0.000	1.000	0.000	1.000	0.000
17	rs4238989[4]	G	C	0.475	0.525	0.451	0.549	0.493	0.507

SNPs highlighted in gray failed various Quality Control criteria and were removed prior to analysis.

[1] SNP was monomorphic or had MAF <0.01 in full sample, case samples, or control samples

[2] SNP was in complete LD ($r^2 \geq 0.9$) with another tag SNP included in the final dataset

[3] SNP failed HWE test (P-value < 0.001) in cases and controls separately. Inbreeding coefficient calculation showed an excess of heterozygotes in both cases and controls

[4] SNP genotyping efficiency was <95%

Table S3A. MDD Combined Dataset Genotype Distributions

SNP	MDD Classification	Genotype Distribution[1]			MAF[2]	HWE[3]
		11	*12*	*22*		
AB047536	Cases	68	273	249	0.347	0.600
	Controls	95	350	318	0.354	0.931
rs228669	Cases	7	85	500	0.084	0.125
	Controls	3	106	667	0.072	0.577
rs228697	Cases	8	134	450	0.127	0.577
	Controls	10	126	640	0.094	0.187
rs17031614	Cases	1	48	543	0.042	0.956
	Controls	0	40	736	0.026	0.461
rs1811399	Cases	27	210	355	0.223	0.563
	Controls	53	272	451	0.244	0.175
rs2117714	Cases	43	254	295	0.287	0.243
	Controls	72	325	379	0.302	0.847
rs4851377	Cases	108	304	179	0.440	0.287
	Controls	186	363	225	0.475	0.097
rs34705978	Cases	19	199	373	0.201	0.222
	Controls	32	216	528	0.180	0.102
rs17717414	Cases	16	182	394	0.181	0.354
	Controls	34	233	509	0.194	0.269
rs2304669	Cases	10	147	435	0.141	0.547
	Controls	15	198	563	0.147	0.617
rs1801260	Cases	45	216	331	0.258	0.242
	Controls	67	303	406	0.282	0.331
rs8192550	Cases	0	41	551	0.035	0.389
	Controls	4	60	712	0.044	0.032
rs70965440	Cases	0	21	571	0.018	0.660
	Controls	0	19	756	0.012	0.730
rs70965441	Cases	1	30	561	0.027	0.375
	Controls	0	45	731	0.029	0.406
rs10830963	Cases	38	235	319	0.263	0.546
	Controls	39	312	425	0.251	0.056
rs1562444	Cases	150	281	161	0.491	0.221
	Controls	203	379	194	0.506	0.521
rs10548381	Cases	6	107	479	0.101	0.993
	Controls	14	121	634	0.097	0.005
rs7137588	Cases	90	257	245	0.369	0.099
	Controls	106	349	321	0.362	0.474

[1]For Genotype distributions; 11-Homozygote (Minor Allele), 12-Heterozygotes, 22- Homozygote (Major Allele)

[2] Minor Allele Frequency in cases or controls

[3] Hardy-Weinberg Equilibrium P-value in cases or controls determined using (--hardy2) option in PLINK

Table S3B. MDD Female-Only Subset Genotype Distributions

SNP	MDD Classification	Genotype Distribution[1]			MAF[2]	HWE[3]
		11	*12*	*22*		
AB047536	Cases	54	233	209	0.344	0.358
	Controls	60	244	209	0.355	0.378
rs228669	Cases	7	72	419	0.086	0.062
	Controls	2	77	444	0.077	0.487
rs228697	Cases	7	116	375	0.131	0.558
	Controls	8	90	425	0.101	0.207
rs17031614	Cases	1	40	457	0.042	0.899
	Controls	0	29	494	0.028	0.514
rs1811399	Cases	23	177	298	0.224	0.613
	Controls	34	180	309	0.237	0.266
rs2117714	Cases	34	214	250	0.283	0.191
	Controls	44	220	259	0.295	0.777
rs4851377	Cases	86	256	155	0.431	0.261
	Controls	119	245	157	0.464	0.214
rs34705978	Cases	16	173	308	0.206	0.159
	Controls	22	143	358	0.179	0.116
rs17717414	Cases	10	151	337	0.172	0.140
	Controls	23	151	349	0.188	0.203
rs2304669	Cases	9	120	369	0.139	0.833
	Controls	10	131	382	0.144	0.750
rs1801260	Cases	39	189	270	0.268	0.463
	Controls	43	204	276	0.277	0.541
rs8192550	Cases	0	30	468	0.030	0.488
	Controls	2	40	481	0.044	0.244
rs70965440	Cases	0	19	479	0.019	0.664
	Controls	0	11	511	0.011	0.808
rs70965441	Cases	1	25	472	0.027	0.281
	Controls	0	30	493	0.029	0.500
rs10830963	Cases	32	198	268	0.263	0.570
	Controls	23	210	290	0.245	0.049
rs1562444	Cases	136	240	122	0.514	0.430
	Controls	120	254	149	0.472	0.557
rs10548381	Cases	6	85	407	0.097	0.515
	Controls	10	85	422	0.102	0.024
rs7137588	Cases	78	213	207	0.370	0.064
	Controls	69	236	218	0.358	0.684

[1]For Genotype distributions; 11-Homozygote (Minor Allele), 12-Heterozygotes, 22- Homozygote (Major Allele)

[2] Minor Allele Frequency in cases or controls

[3] Hardy-Weinberg Equilibrium P-value in cases or controls determined using (--hardy2) option in PLINK

Table S3C. MDD Male-Only Subset Genotype Distributions

SNP	MDD Classification	Genotype Distribution[1]			MAF[2]	HWE[3]
		11	*12*	*22*		
AB047536	Cases	14	40	40	0.362	0.447
	Controls	35	106	109	0.352	0.265
rs228669	Cases	0	13	81	0.069	0.471
	Controls	1	29	223	0.061	0.956
rs228697	Cases	1	18	75	0.106	0.945
	Controls	2	36	215	0.079	0.718
rs17031614	Cases	0	8	86	0.043	0.667
	Controls	0	11	242	0.022	0.724
rs1811399	Cases	4	33	57	0.218	0.776
	Controls	19	92	142	0.257	0.449
rs2117714	Cases	9	40	45	0.309	0.980
	Controls	28	105	120	0.318	0.489
rs4851377	Cases	22	48	24	0.489	0.833
	Controls	67	118	68	0.498	0.285
rs34705978	Cases	3	26	65	0.170	0.840
	Controls	10	73	170	0.184	0.542
rs17717414	Cases	6	31	57	0.229	0.527
	Controls	11	82	160	0.206	0.904
rs2304669	Cases	1	27	66	0.154	0.328
	Controls	5	67	181	0.152	0.676
rs1801260	Cases	6	27	61	0.207	0.220
	Controls	24	99	130	0.291	0.419
rs8192550	Cases	0	11	83	0.059	0.547
	Controls	2	20	231	0.047	0.047
rs70965440	Cases	0	2	92	0.011	0.917
	Controls	0	8	245	0.016	0.798
rs70965441	Cases	0	5	89	0.027	0.791
	Controls	0	15	238	0.030	0.627
rs10830963	Cases	6	37	51	0.261	0.837
	Controls	16	102	135	0.265	0.574
rs1562444	Cases	28	41	25	0.516	0.219
	Controls	54	125	74	0.461	0.929
rs10548381	Cases	0	22	72	0.117	0.199
	Controls	4	36	212	0.087	0.100
rs7137588	Cases	12	44	38	0.362	0.894
	Controls	37	113	103	0.370	0.509

[1]For Genotype distributions; 11-Homozygote (Minor Allele), 12-Heterozygotes, 22- Homozygote (Major Allele)

[2] Minor Allele Frequency in cases or controls

[3] Hardy-Weinberg Equilibrium P-value in cases or controls determined using (--hardy2) option in PLINK

Table S3D. Specific Genotype Distribution Definitions for all Markers Analyzed

CHR	SNP	Gene	Genotype Distribution[1] *11*	*12*	*22*
1	AB047536	PER3	5/5	4/5	4/4
1	rs228669	PER3	AA	AG	GG
1	rs228697	PER3	GG	GC	CC
1	rs17031614	PER3	AA	AG	GG
2	rs1811399	NPAS2	GG	GT	TT
2	rs2117714	NPAS2	CC	CT	TT
2	rs4851377	NPAS2	CC	CT	TT
2	rs34705978	NPAS2	TT	TC	CC
2	rs17717414	NPAS2	GG	GA	AA
2	rs2304669	PER2	GG	GA	AA
4	rs1801260	CLOCK	CC	CT	TT
4	rs8192550	MTNR1A	GG	GA	AA
11	rs70965440	ARNTL	GG	GT	TT
11	rs70965441	ARNTL	CC	CA	AA
11	rs10830963	MTNRIB	GG	GC	CC
11	rs1562444	MTNRIB	AA	AG	GG
12	rs10548381	ARNTL2	TT	TA	AA
12	rs7137588	ARNTL2	CC	CG	GG

[1]For Genotype distributions; 11-Homozygote (Minor Allele), 12-Heterozygotes, 22- Homozygote (Major Allele)

Table S4. Summary of non-significant Chi-square analysis results for association with MDD in Combined Dataset

CHR	SNP	GENE	TEST[1]	COMBINED PVALUE[2]	FEMALE PVALUE[3]	MALE PVALUE[4]
1	AB047536	PER3	GENO	0.8737	-	-
1	AB047536	PER3	ALLELIC	0.6949	-	-
1	rs228669	PER3	GENO	0.207*	-	-
1	rs228669	PER3	ALLELIC	0.266	-	-
2	rs1811399	NPAS2	GENO	0.205	-	-
2	rs1811399	NPAS2	ALLELIC	0.208	-	-
2	rs2117714	NPAS2	GENO	0.412	-	-
2	rs2117714	NPAS2	ALLELIC	0.393	-	-
2	rs17717414	NPAS2	GENO	0.260	-	-
2	rs17717414	NPAS2	ALLELIC	0.382	-	-
2	rs2304669	PER2	GENO	0.900	-	-
2	rs2304669	PER2	ALLELIC	0.666	-	-
4	rs8192550	MTNR1A	GENO	0.207*	-	-
4	rs8192550	MTNR1A	ALLELIC	0.224	-	-
11	rs70965441	ARNTL	GENO	0.465*	-	-
11	rs70965441	ARNTL	ALLELIC	0.758	-	-
11	rs10830963	MTNR1B	GENO	0.541	-	-
11	rs10830963	MTNR1B	ALLELIC	0.499	-	-
11	rs1562444	MTNR1B	GENO	0.656	-	-
11	rs1562444	MTNR1B	ALLELIC	0.434	-	-
12	rs10548381	ARNTL2	GENO	0.263	-	-
12	rs10548381	ARNTL2	ALLELIC	0.753	-	-
12	rs7137588	ARNTL2	GENO	0.690	-	-
12	rs7137588	ARNTL2	ALLELIC	0.682	-	-

[1] Describes the type of Chi-Square test performed; genotypic or allelic
[2] P-values are from Chi-Square test performed in the combined dataset
*Fisher's Exact P-value or One-sided Fisher's Exact P-value shown

Table S5. Prevalence-based Association Test (PRAT) Analysis in Combined and Sex-Separated Subsets

CHR	SNP	GENE	FULL CASE PVALUE[1]	FULL CONTROL PVALUE[1a]	FEMALE CASE PVALUE[2]	FEMALE CONTROL PVALUE[2a]	MALE CASE PVALUE[3]	MALE CONTROL PVALUE[3a]
1	AB047536	PER3	0.774	0.939	0.703	0.488	0.773	0.537
1	rs228669	PER3	0.116	0.577	0.158	0.539	0.717	0.904
1	rs228697	PER3	**0.006**	0.103	**0.033**	0.094	0.488	0.654
1	rs17031614	PER3	**0.012**	0.443	0.079	0.567	0.104	1.000
2	rs1811399	NPAS2	0.332	0.109	0.636	0.148	0.520	0.343
2	rs2117714	NPAS2	0.324	0.827	0.471	0.85	0.962	0.369
2	rs4851377	NPAS2	0.109	**0.033**	0.120	0.082	0.965	0.243
2	rs34705978	NPAS2	0.129	**0.026**	0.052	**0.032**	0.939	0.503
2	rs17717414	NPAS2	0.386	0.110	0.210	0.071	0.605	0.917
2	rs2304669	PER2	0.754	0.616	0.876	0.689	0.618	0.760
4	rs1801260	CLOCK	0.275	0.615	0.759	0.558	0.081	0.717
4	rs8192550	MTNR1A	0.465	0.090	0.298	0.252	0.759	0.101
11	rs70965440	ARNTL	0.209	0.962	0.096	0.937	0.742	0.564
11	rs70965441	ARNTL	0.666	0.176	0.672	0.609	1.000	0.522
11	rs10830963	MTNRIB	0.787	0.196	0.678	0.149	0.984	0.513
11	rs1562444	MTNRIB	0.444	0.677	0.068	0.591	0.168	0.906
12	rs10548381	ARNTL2	0.977	0.062	0.936	0.174	0.142	**0.008**
12	rs7137588	ARNTL2	0.427	0.795	0.296	0.887	0.969	0.364

Significant P-values are shown in bold (p≤0.05). P-values shown are permutation p-values corrected for multiple testing.

[1,1a] P-values are from analysis of the case or control populations of the Combined dataset, respectively.

[2,2a] P-values are from analysis of the case or control populations of the Female-only dataset, respectively.

[3,3a] P-values are from analysis of the case or control populations of the Male-only dataset, respectively.

Supplementary Information for Shi et al.:

Molecular Analyses of Circadian Gene Variants Reveal Sex-dependent Links Between Depression and Clocks

Figure S1, A priori power calculations for various effect sizes. Sample size refers to number of cases. Effect size is measured as the odds ratio from a logistic regression analysis of the relationship between genotype at a SNP with minor allele frequency 0.2 and susceptibility to MDD. Estimated power for various effect sizes is indicated by colored lines.

Figure S2, related to Figure 1. Functional analysis of *hClock* SNP in cell cultures.

(A) Cos-1 (monkey kidney) cells were transfected with P_{SV40}::FLuc::3' UTR.
(B) U2OS (human osteosarcoma) were transfected with P_{SV40}::FLuc::3' UTR.

Methods are as in Figure 1. Levels of firefly luciferase activity were normalized with the *Renilla* luciferase reporter (pCMV-Rluc). The activity in samples transfected with the reporter constructs containing the common 3'UTR allele (pCI-Luc-T or pSV40-luc-T) was set as 1.0. Results are shown as mean±SEM of two independent experiments (left panel, total n = 12; right panel, total n = 8). *p < 0.05 by two-tail unpaired t-test. The P_{SV40}::FLuc::3'UTR reporter was kindly provided by Dr. Malcolm von Schantz.

Figure S3 related to Tables 1 and 2. Statistical Analysis Flow Chart.

Figure S4, related to Figure 1. *hClock* expression is modulated by dexamethasone and the rs1801260 SNP. Schematic shows a glucocorticoid response element (GRE) in the SNP region of 3' UTR of the *hClock* gene. In this figure, the minor allele (rs1801260 SNP) is "C" (indicated by yellow font) and the major allele is "T" (indicated by green font). The consensus motifs of activated vs. repressed GREs are based on the analyses of Kuo and coworkers,[1] and these portions of the figure are modified from that publication.

Figure S5, related to Figure 2. Degradation of hPER3 protein is slowed by the rs228697 variant. Immunoblotting was used to assess the transfected expression of Flag-hPER3 in HEK 293 after CHX treatment (GAPDH served as loading control, WT = wild-type, MT = rs228697 variant). Two independent immunoblots are shown in panel A, and quantification of Flag-hPER3 protein expression normalized with GAPDH for each experiment is shown in panel B (for both Flag-hPer3-WT and Flag-hPer3-MT, the expression at the first time-point {0.1h} after CHX treatment was set as 1).

Figure S6, related to Figure 2. Interaction between PER2 and PER3 in HEK293 cells. HEK293 cells were co-transfected with Flag-h*Per3* with or without the rs228697 SNP (wildtype, WT; or rs228697 variant/mutation, MT) and m*Per2*-V5 (mP2). Immunoprecipitates were prepared using a limiting amount of anti-Flag antibody and the immunoprecipitates (IP) and original cell lysates (IB) were separated by electrophoresis and immunoblotted with either anti-Flag (for hPER3) or anti-V5 (for mPER2). **A**. Two independent immunoblot experiments (Exp 1/2). The input level of the cell lysates and of the anti-Flag antibody for IP in experiment 1 were approximately 3X more than those in experiment 2. GAPDH was served as loading control. **B**. Upper panel (IP): Quantification of the mPER2-V5 protein that was co-immunoprecipitated with Flag-hPER3

antibody. The amount of hPER3WT pull-down was normalized to 1.0. Lower panel (IB): Amount of hPER3WT (native hPER3) and hPER3MT (rs228697 variant/mutation) present in the cell lysates.

Table S1A. Demographic description of MDD Study Participants

Table S1B. Demographic description of MDD Study Participants by gender

Table S2. Summary of all Genotyped Markers

Table S3A. MDD Combined Dataset Genotype Distributions

Table S3B. MDD Female-Only Subset Genotype Distributions

Table S3C. MDD Male-Only Subset Genotype Distributions

Table S3D. Specific Genotype Distribution Definitions for all Markers Analyzed

Table S4. Summary of non-significant Chi-square analysis results for association with MDD in Combined Dataset

Table S5. Prevalence-based Association Test (PRAT) Analysis in Combined and Sex-Separated Subsets

Supplemental Materials and Methods

Participants

In an effort to aid in the advancement of genetic research directed at severe neuropsychiatric disorders, the National Institute of Mental Health (NIMH) funded the Human Genetics Initiative (HGI, see acknowledgements below).[2] The HGI compiled a database of clinically diagnosed MDD pedigrees, including MDD cases and affected relatives (Depression 2.0, NIMH Major Depression Genetics Initiative). All participants were administered the Diagnostic Interview for Genetic Studies (DIGS v2.0) by trained interviewers. Probands who self-reported as Caucasian and carried a clinical diagnosis of subtype 1 MDD, also termed melancholia, were selected from this dataset for our study. We identified participants with past or current alcohol or drug abuse based on positive answers to specific questions from the DIGS v2.0: (i) the subject experienced at least 3 positive symptoms of abuse occurring in the same 12 month period; OR (ii) two of the positive symptoms persisted for over a month or occurred over a longer period of time. In the DIGS v2.0, positive symptoms are described for alcohol abuse in starred (*) questions 13-33 in the 'Alcohol Abuse and Dependence' section and for drug abuse in starred (*) questions 19-33 in the 'Drug Abuse and Dependence' section.

The HGI also collected a broad-based control sample to aid in case-control association studies focused on neuropsychiatric disorders. These controls were phenotyped using an online

lifetime version of the Composite Instrument for Diagnostic Interviewing Short Form (CIDI-SF); an online clinical self-report assessment of MDD and several other psychiatric conditions that have been previously described.[3] Because this dataset was to function as a control database for a wide number of disorders, individuals who indicated the presence of mental disorders or substance abuse were not excluded from the HGI control dataset. Investigators were advised to screen the HGI control sample prior to analysis to ensure that none of the controls that they included in their sample possessed their disorder of interest. In our analyses all controls whose responses indicated the presence of lifetime MDD or self-reported as other than Caucasian were excluded. The presence of MDD in the HGI control dataset was identified by either (i) self-report of clinical diagnosis of MDD or (ii) affirmation of being depressed for two weeks or more in a row (questions A1A, A1B) and experiencing a total of 5 symptoms in the MDD category (questions A1A, A1B, A2, A3, A4). Controls were also excluded if they declined to answer a survey question that was critical to determining MDD diagnosis. Participants with past or current alcohol or drug abuse were also identified in the HGI dataset. The presence of alcohol abuse was indicated by affirmation that (i) drinking interfered with work, school, or home life (question G2) at least 3-5 times (question G2A) and (ii) participants were either under the influence of alcohol in a situation where they could get hurt (question G3) or the participant had emotional or psychological problems from using alcohol (question G4). The presence of drug abuse was indicated by affirmation that (i) the participant used a drug either without a doctor's prescription, in larger amounts than prescribed, or for longer than prescribed (questions H1_1-H1_9) and (ii) drug use interfered with work, school, or home life (question H3) at least 3-5 times (question H3) and (iii) participants were either under the influence of drugs in a situation where they could get hurt (question H4) or the participant had emotional or psychological problems from using drugs (question H5).

Informed consent was obtained for all HGI dataset and NIMH Major Depression Genetics Initiative participants prior to DNA collection.

Genotyping

DNA samples from the HGI and NIMH Major Depression Genetics datasets were genotyped for 32 polymorphisms within circadian clock genes that are common in the Caucasian population and have potential functional significance based on well-characterized roles in regulating circadian rhythms and/or sleep disorders.[4,5]

Single Nucleotide polymorphism screening and genotyping. All SNPs (except the variable number of tandem repeat in exon 18 of *hPer3*, AB047536) were genotyped by Sequenom screening (Sequenom Inc. San Diego Ca). DNA samples were loaded in 384-well plates and subjected to PCR primer extension. The single base extension products were detected with a Mass Array MALDI-TOF mass spectrometer on a Sequenom iPLEX genotyping platform. The sequence difference at the single nucleotide level was detected as an allele-specific difference in mass between extension products. The difference in mass was correlated to a specific genotype. The call rate threshold for all the SNPs analyzed by Sequenom was at least 95%.

Variable Number of Tandem Repeat polymorphism screening and genotyping. PCR genotyping for the *hPer3* VNTR (AB047536) utilized primers as previously described.[6] Platinum PCR Supermix (Invitrogen, Carlsbad, CA, USA) was used to produce the 475 vs. 550 bp amplicon from genomic DNA using the following parameters: 95°C for 90 sec, then 35 cycles of 95°C for 30 sec, 55°C for 30 sec and 72°C for 30 sec, followed by a final extension at 72°C for 7 min and a 4°C hold. After separation by electrophoresis in a 2% agarose gel and visualized by ethidium bromide staining, the PCR products distinguished between the 5-repeat allele (550 bp) and the

4-repeat allele (475 bp).

Association Analyses

After genotyping, the samples were assessed for genotyping efficiency (SNP and sample-level) and deviation from Hardy-Weinberg Equilibrium (HWE) as measures of quality control (QC). The following QC exclusion criteria were applied to the our dataset: (1) SNP genotyping efficiency < 95%, and (2) sample genotyping efficiency < 95%, HWE test p-value < 0.001. All QC testing was performed using PLINK.[7] After quality control, 18 genetic variants remained for analysis in our dataset. Prior to beginning analysis, we performed power calculations using PS Power[8] to determine whether our study was adequately powered to detect common variants with modest effects on MDD. A priori power calculations indicated that our study was well powered (≥ 80%) to detect common variants (minor allele frequency ≥ 0.2) with modest effect sizes, and α= 0.5 (Figure S1).

To assess statistical association between the analyzed SNPs and MDD, single locus analyses were performed in the combined dataset (n = 592 cases, 776 controls). Allelic and genotypic Chi-square analyses were performed, using PLINK.[7] Where appropriate, Fisher's Exact test of association was performed. To investigate the possibility of potentially differing effects of the analyzed SNPs on MDD between the genders, sex separated analyses were also performed (males, n= 94 cases, 253 controls; females, n = 498 cases, 523 controls) for all markers that showed a p-value < 0.2 in Chi-square genotypic or allelic tests in the full dataset. Correction for multiple testing of Chi-square p-values was performed using False Discovery Rate (FDR) (q = 0.1).[9]

An alternative single locus analysis, Prevalence-based Association testing (PRAT), based on the principles of HWE has been shown in simulation studies to be more powerful than Chi-square testing under some genetic models and was also performed to provide independent evidence for association.[10] PRAT uses an estimated population allele frequency to generate expected genotype frequencies, taking into account the population prevalence of the selected disease. These expected genotype frequencies are then used to calculate expected numbers of individuals in each genotype class for the cases and controls separately, under the assumption of no association between SNP and phenotype, and Chi-square statistics are generated from these. The resulting PRAT p-value is then assessed for cases and controls independently. Significant PRAT p-values for either cases or controls, or both, are indicative of an association.[10] Prevalence of MDD was set at 7% for PRAT analysis as an approximation of the Caucasian population prevalence of MDD (NIMH statistic 2005), and 1000 permutations of affection status were performed to correct for multiple testing. Simulation data has demonstrated that minor misspecification of prevalence rates do not affect results significantly.[10] PRAT was performed in the combined and sex separated datasets as a part of the PLATO software package.

Therefore, to confirm significant SNPs by a different and methodologically independent analysis, we evaluated the associations between MDD and our assayed genetic variants using Prevalence-based Association Testing (PRAT)[10] (Table S5). PRAT analyses in the full dataset provided additional evidence for the significant association between four variants previously identified using χ^2 analyses (rs228697 and rs17031614 in hPER3, and rs485133 and rs34705978 in hNPAS2) (Table S5). Additionally, PRAT analysis in the male subset revealed a novel association between MDD and a single variant in Arntl2, rs7137588 (p-value = 0.008) (Table S5). While none of the SNPs reached significance at the p ≤ 0.05 level in the male case-

only dataset in the PRAT analysis, rs1801260 of *hClock* was the only SNP in the male case-only subset that was marginally significant (p = 0.081, Table S5).

To assess effect size of statistically significant SNPs discovered using the previous two methods, logistic regression, and where appropriate Exact logistic regression, using an additive model was performed using the STATA version 10.0 software package (StataCorp, College Station, TX). In cases where sample size was < 5 in any genotypic category, but Exact logistic regression was computationally infeasible, standard logistic regression using a dominant model for the risk allele was employed.

Functional Analyses

Construction of luciferase reporters and expression vectors: The P_{SV40}::FLuc::3'UTR firefly luciferase reporter for the 3'UTR of *hClock* (containing the major allele T) was provided by Dr. Malcolm von Schantz.[11] To create a minor allele reporter, a T to C mutation at position 3111 (rs1801260) was introduced by site-directed mutagenesis (Stratagene) and verified by sequencing. To create P_{Clock}::FLuc plasmids so that the luciferase expression is under the control of the endogenous *hClock* promoter, a fragment extending from the *hClock* upstream region (-1908 from the transcription start site) to the first intron (+101 from the transcription start site) was amplified by PCR from human cell genomic DNA and was used to replace the SV40 promoter of the P_{SV40}::FLuc-T/C plasmids. The *hPer3* expression plasmid under the control of the CMV promoter (P_{CMV}::hPer3) was provided by Dr. Joon-Kyu Lee.[12] A G to C mutation (rs228697) in the coding region of *hPer3* was introduced to change the proline at position 856 to an alanine residue by site-directed mutagenesis (Stratagene) that was verified by sequencing.

Cell culture and functional assay experiments: BMAL1/CLOCK transactivation of E-box containing promoters (and PER3 repression thereof) was assessed by a transient transfection assay.[13,14] Cell lines {HEK-293T, HEK-293 (ATCC), HepG2 (a gift from Dr. Richard O'Brien, Vanderbilt Medical Center), COS-1 (a gift from Dr. Stephen Brandt, Vanderbilt Medical Center)} were maintained at 37°C, 5% CO2 for 24 h in a 24-well plate in Dulbecco's modified Eagle's medium supplemented with 10% fetal bovine serum and 1% penicillin, streptomycin and glutamine solution. All four cell lines have been authenticated within the past two years and tested for mycoplasma contamination.

Because our association analyses indicated sex-dependent effects, we also wanted to test cell culture lines that were originally derived from both males and females. The sex of the cell culture lines we tested is as follows:

Female derivation: HEK293T, U2OS; Male derivation: HepG2, COS-1.

Methodology:

1. Using the P_{Clock}::FLuc::3'UTR or P_{SV40}::FLuc::3'UTR reporters to characterize the expression levels affected by the Clock SNP (rs1801260), the cells were transiently transfected with one of the reporters (T or C allele) and a control vector (P_{CMV}::Rluc) using Lipofectamine 2000 (Invitrogen). After transfection (24 h), the cells were harvested and the luciferase activity was measured using the Dual Luciferase Reporter Assay kit (Promega).

2. To determine the sensitivity of the P_{Clock}::FLuc::3'UTR or P_{SV40}::FLuc::3'UTR reporters containing the Clock SNP (rs1801260) in response to glucocorticoid stimuli, HepG2 cells were transfected with the same of amount of reporter plasmid and the P_{CMV}::Rluc control vector. After

transfection (22 h), 0, 50, or 500 nM dexamethasone dissolved in ethanol was added to cells (final ethanol concentration 0.1%). Two hours after dexamethasone treatment, the cells were harvested for dual luciferase measurement.

3. To determine the influence of the *hPer3* SNP (rs228697) on E-box transcriptional activation, cells were transfected with plasmids encoding BMAL1, mCLOCK, reporters consisting of two different E-box containing promoters fused to firefly luciferase ($P_{PK2.8}$::FLuc or P_{AVP}::FLuc), P_{CMV}::hPer3 (major G or minor C allele), and the P_{CMV}::Rluc control vector. These plasmids were transfected into HEK-293 cells using the Lipofectamine 2000 (Invitrogen). The total amount of DNA constructs for each transfection experiment was kept equal by adding appropriate mock vectors. After transfection (24 h), the cells were harvested and luciferase activity was measured by the Dual Luciferase Reporter Assay kit (Promega). For these expression studies (Figs. 1, 2, S2), data are expressed as mean ± SEM. Statistical analyses were performed by two-tail unpaired T test or one-way ANOVA with Turkey post hoc as indicated.

4. To measure the effect of hPER3 expression on circadian rhythms in cell cultures (Figure 2C/D/E), Rat-1 cells were co-transfected with *hPeriod3* (native hPER3 = hP3WT; the rs228697 SNP-containing hPER3 = hP3MT) under the CMV promoter of pCDNA3, and with a P_{Bmal1}::FLuc reporter using Fugene 6 reagent (Roche). The control cells were co-transfected with a similar amount of pCDNA3.1 empty vector (Invitrogen) and the P_{Bmal1}::FLuc reporter. One day after transfection, cells were synchronized by 100 nM Dexamethasone for 2 h and then luminescence emission was monitored in a Lumicycle apparatus (Actimetrics). The *in vitro* rhythms were analyzed using LumiCycle analysis software (Actimetrics, version 2.31).

5. In order to examine the influence of a proline to alanine mutation at residue 856 of human Per3 on the degradation rate of PER3 and PER2 proteins (Figure S5), HEK293 cells were transfected with Flag-*hPer3* plasmids with or without the rs228697 SNP (wildtype, WT; or rs228697 mutation, MT) by Fugene 6 transfection reagent (Roche). The DNA constructs were under the control of the CMV promoter. One day later, cycloheximide (CHX, 80μg/ml, Sigma) was added to cells, and cells were harvested 0.1, 3, 5, 8, and 10 hours after CHX treatment. Anti-Flag antibody (Sigma F3165), anti-V5 antibody (Invitrogen R960), and anti-GAPDH antibody (Abcam ab9484) were used to probe protein expression.

6. To test the physical interaction between hPER3 and mPER2 proteins (Figure S6), HEK293 cells were co-transfected with Flag-h*Per3* with or without the rs228697 SNP (wildtype, WT; or rs228697 mutation, MT) and with m*Per2*-V5 plasmids by Fugene 6 transfection reagent (Roche). Both DNA constructs were under CMV promoter control. The cell extracts were immunoprecipitated by adding anti-Flag antibody (Sigma F7425). Precipitated proteins and cell extracts from transfected cells (e.g., for experiment#2 in Figure S6, 15% of the cell extract was used for the "IB input" sample and 85% of the extract was treated with antibody and constitutes the "IP" sample) were examined by electrophoresis in 4-12% Bis-Tris gels (Life Technologies) and immunoblotting. Anti-Flag antibody (Sigma F3165 or F7425), anti-V5 antibody (Invitrogen R960) were used to probe Flag tagged hPER3, and V5 tagged mPER2 respectively. Densitometric analyses were performed using Image J software (NIH).

Supplemental Acknowledgements

Acknowledgment for Depression Sample Biomaterials and Clinical Data:
Data and biomaterials were collected in six projects that participated in the National Institute of Mental Health (NIMH) Genetics of Recurrent Early-Onset Depression (GenRED) project. From

1999-2003, the Principal Investigators and Co-Investigators were: New York State Psychiatric Institute, New York, NY, R01 MH060912, Myrna M. Weissman, Ph.D. and James K. Knowles, M.D., Ph.D.; University of Pittsburgh, Pittsburgh, PA, R01 MH060866, George S. Zubenko, M.D., Ph.D. and Wendy N. Zubenko, Ed.D., R.N., C.S.; Johns Hopkins University, Baltimore, R01 MH059552, J. Raymond DePaulo, M.D., Melvin G. McInnis, M.D. and Dean MacKinnon, M.D.; University of Pennsylvania, Philadelphia, PA, RO1 MH61686, Douglas F. Levinson, M.D. (GenRED coordinator), Madeleine M. Gladis, Ph.D., Kathleen Murphy-Eberenz, Ph.D. and Peter Holmans, Ph.D. (University of Wales College of Medicine); University of Iowa, Iowa City, IW, R01 MH059542, Raymond R. Crowe, M.D. and William H. Coryell, M.D.; Rush University Medical Center, Chicago, IL, R01 MH059541-05, William A. Scheftner, M.D. Rush-Presbyterian.

Acknowledgment for Control Sample Biomaterials and Clinical Data:
Control subjects from the National Institute of Mental Health Schizophrenia Genetics Initiative (NIMH-GI), data and biomaterials were collected by the "Molecular Genetics of Schizophrenia II" (MGS-2) collaboration. The investigators and coinvestigators were: ENH/Northwestern University, Evanston, IL, MH059571, Pablo V. Gejman, M.D. (Collaboration Coordinator; PI), Alan R. Sanders, M.D.; Emory University School of Medicine, Atlanta, GA,MH59587, Farooq Amin, M.D. (PI); Louisiana State University Health Sciences Center; New Orleans, Louisiana, MH067257, Nancy Buccola APRN, BC, MSN (PI); University of California-Irvine, Irvine, CA, MH60870, William Byerley, M.D. (PI); Washington University, St. Louis, MO, U01, MH060879, C. Robert Cloninger, M.D. (PI); University of Iowa, Iowa, IA,MH59566, Raymond Crowe, M.D. (PI), Donald Black, M.D.; University of Colorado, Denver, CO, MH059565, Robert Freedman, M.D. (PI); University of Pennsylvania, Philadelphia, PA, MH061675, Douglas Levinson M.D. (PI); University of Queensland, Queensland, Australia, MH059588, Bryan Mowry, M.D. (PI); Mt. Sinai School of Medicine, New York, NY,MH59586, Jeremy Silverman, Ph.D. (PI). In addition, cord blood samples were collected by V L Nimgaonkar's group at the University of Pittsburgh, as part of a multi- institutional collaborative research project with J Smoller, MD DSc and P Sklar, MD PhD (Massachusetts General Hospital) (grant MH 63420).

Supplemental References

1 Kuo T, Lew MJ, Mayba O, Harris CA, Speed TP et al. Genome-wide analysis of glucocorticoid receptor-binding sites in myotubes identifies gene networks modulating insulin signaling. *Proc Natl Acad Sci U S A* 2012; **109:** 11160-11165.

2 Levinson DF, Zubenko GS, Crowe RR, DePaulo RJ, Scheftner WS et al. Genetics of recurrent early-onset depression (GenRED): design and preliminary clinical characteristics of a repository sample for genetic linkage studies. *Am J Med Genet B Neuropsychiatr Genet* 2003; **119:** 118-130.

3 Kessler RC, Andrews G, Mroczek D, Üstün TB, Wittchen HU. The World Health Organization Composite International Diagnostic Interview Short Form (CIDI-SF). *International Journal of Methods in Psychiatric Research* 1998 ; **7:** 171-185.

4 Ciarleglio CM, Ryckman k, Servick SV, Hida A, Robbins S et al. Genetic Differences in Human Circadian Clock Genes Among Worldwide Populations. *J Biol Rhythms* 2008; **23:** 330-340.

5 Gamble KL, Motsinger-Reif AA, Hida A, Borsetti HM, Servick SV et al. Shift Work in Nurses: Contribution of Phenotypes and Genotypes to Adaptation. PLoS One 2011; **6:** e18395.

6 Ebisawa T, Uchiyama M, Kajimura N, Mishima K, Kamei Y. Association of structural polymorphisms in the human period3 gene with delayed sleep phase syndrome. *EMBO Rep* 2001; **2:** 342-346.

7 Purcell S, Neale B, Todd-Brown K, Thomas L, Ferreira MA et al. PLINK: a tool set for whole-genome association and population-based linkage analyses. *American journal of human genetics* 2007 ; **81:** 559-575.

8 Dupont WD, Plummer W. PS power and sample size program available for free on the Internet. Controlled Clin Trials 1997; **18**:274.

9 Benjamini N, Hochberg Y. Controlling the false discovery rate: a practical and powerful approach to multiple testing. *Journal of the Royal Statistical Society, Series B (Methodological)* 1995 ; **57:** 289-300.

10 Ryckman KK, Jiang L, Li C, Bartlett J, Haines JL et al. A prevalence-based association test for case-control studies. *Genet Epidemiol* 2008; **32:** 600-605.

11 Robilliard DL, Archer SN, Arendt J, Lockley SW, Hack LM et al. The 3111 Clock gene polymorphism is not associated with sleep and circadian rhythmicity in phenotypically characterized human subjects. *J Sleep Res* 2002; **11:** 305-312.

12 Im JS, Jung BH, Kim SE, Lee KH, Lee JK. Per3, a circadian gene, is required for Chk2 activation in human cells. *FEBS Lett* 2010; **584:** 4731-4734.

13 Gekakis N, Staknis D, Nguyen HB, Davis FC, Wilsbacher LD et al. Role of the CLOCK protein in the mammalian circadian mechanism. *Science* 1998; **280:** 1564-1569.

14 Kume K, Zylka MJ, Sriram S, Shearman LP, Weaver DR et al. mCRY1 and mCRY2 are essential components of the negative limb of the circadian clock feedback loop. *Cell* 1999; **98:** 193-205.
